# Behavioural phase transitions in the migratory locust, *Locusta migratoria*, are related to changes in the gut bacterial composition

**DOI:** 10.1093/ismeco/ycag009

**Published:** 2026-01-15

**Authors:** Jaeha Kim, Takumi Murakami, Atsushi Toyoda, Hiroshi Mori

**Affiliations:** Department of Informatics, National Institute of Genetics, 1111 Yata, Mishima, Shizuoka 411-8540, Japan; School of Life Science and Technology, Institute of Science Tokyo, 2-12-1 Ookayama, Meguro-ku, Tokyo 152-8550, Japan; Advanced Genomics Center, National Institute of Genetics, 1111 Yata, Mishima, Shizuoka 411-8540, Japan; Department of Informatics, National Institute of Genetics, 1111 Yata, Mishima, Shizuoka 411-8540, Japan; Advanced Genomics Center, National Institute of Genetics, 1111 Yata, Mishima, Shizuoka 411-8540, Japan

**Keywords:** shotgun metagenome, 16S rRNA, *locusta migratoria*behavioral phase transition, gut bacterial composition, gut-brain, symbiosis

## Abstract

*Locusta migratoria* is a grasshopper species that can change its behaviour from solitary to gregarious. Previous studies have implicated metabolites such as serotonin and dopamine in the regulation of behavioural transition in this species. While many studies using cultured microbes have demonstrated that some microbes harbor the neuroactive metabolic potential of these neurotransmitters, the association between microbial community composition and phase transition remains poorly understood. Here, we employed 16S rRNA gene amplicon sequencing and shotgun metagenomic sequencing analyses to compare the composition of gut microbial communities of *L. migratoria* in different behavioural phases. We found that *Serratia ureilytica* was enriched in the gut of gregarious individuals in contrast to the decreased presence of *Klebsiella aerogenes,* one of the most abundant taxa in wild individuals. The gut microbiome of gregarious individuals was functionally characterised by enriched kynurenine and tryptophan synthesis pathways, and by reduced representation of GABA, indole, and dopamine metabolism pathways compared with that of solitary individuals. These compositional changes were consistent with the enrichment of *S. ureilytica* and depletion of *K. aerogenes*, which possess the corresponding genes. In particular, the genes for kynurenine synthesis encoded by *S. ureilytica* specific to the gregarious phase, are known to be involved in the tryptophan production and are associated with reduced serotonin synthesis. These results highlight a distinct shift in both the taxonomic and functional composition of the gut microbiome across behavioural phases and suggest a potential microbial contribution to the behavioural changes of *L. migratoria*.

## Introduction

The density-dependent phase transition of grasshoppers, commonly referred to as the “locust” phenomenon, threatens a stable food supply by causing extensive agricultural damage, particularly in developing regions [1–3]. Two species in the family Acrididae, *Schistocerca gregaria* [4] and *Locusta migratoria* [5], have been widely reported to exhibit density-dependent gregarious phase change in natural populations [6, 7]. When entering the gregarious phase at high population densities, these species form large swarms that migrate over long distances. This phase is also characterised by the emergence of cannibalistic behaviour [8–11] and exhibited morphological changes, including alterations in body colouration and the shape of body parts [12–14].

A pivotal study in *Sanicula gregaria* suggested that increased tactile stimulation of the legs is strongly associated with behavioural transitions to the gregarious phase [15]. Since then, it has become widely accepted that such behavioural changes are triggered by physical stimulation and stress resulting from frequent contact at high population densities. Subsequent studies have further shown that elevated serotonin levels correlate with gregarious behaviour in *S. gregaria* [16], providing a biochemical basis for the phase transition mechanism. Interestingly, a species-specific divergence in the role of serotonin has been reported: while it promotes gregarious behaviour in *S. gregaria*, it induces solitary behaviour in *L. migratoria* [17], suggesting potential mechanistic differences in phase transition between these phylogenetically distant species [18]. These density-dependent phenotypic changes exemplify phenotypic plasticity, indicating that they are reversible and not associated with alterations in the genome sequence.

However, phenotypic plasticity alone does not fully explain the complexity of the phase transition. Notably, phenol and its derivative guaiacol—pheromonal compounds known to promote aggregation in gregarious individuals—have been reported to be synthesised not only by the host but also by *Pantoea agglomerans*, a gut microbe isolated from *S. gregaria* faeces [19]. This finding suggests that gut microbes may play an important role in gregarious phase transition. Supporting this hypothesis, 16S rRNA gene amplicon sequencing has revealed compositional differences in the gut microbes between gregarious and solitary *S. gregaria* individuals [20]. Further study has proposed that horizontal transmission of the gut microbes among *S. gregaria* individuals contributes to these differences [21].

Additional evidence for microbial involvement in phase transition comes from studies on the microsporidian parasite *Paranosema locustae*, which suppresses the gregarious phase transition in *L. migratoria* [22]. A subsequent study revealed that this suppression is accompanied by a reduction in gut bacterial diversity [23], implicating the gut microbes may influence the gregarious phase transition in *L. migratoria*. In humans and mice, accumulating evidence has shown that gut microbes can produce neurotransmitters—such as dopamine, serotonin, and γ-aminobutyric acid (GABA)—that influence host neural signalling and behaviour through the gut–brain axis [24]. Several studies in *Drosophila* [25–27], honeybee [28–30] have also highlighted the potential roles of microbial metabolites in modulating host behaviour in insects, as further reviewed in [31, 32]. Research on other phytophagous insects, including termites [33–35] and leafcutter ants [36], has also shown that gut microbiota contribute substantially to digestive processes and may influence the production of chemical cues involved in nestmate recognition [37]. Together, these findings imply a potential link between gut microbes and behavioural phase change in locust grasshoppers.

Despite such progress in several animal taxa, our understanding of how gut microbes affect the physiology and behaviour of locust grasshoppers remains limited. While several studies have described the taxonomic composition of locust gut microbes [19–23, 38], none have investigated whether these microbes encode genes involved in the synthesis or degradation of neurotransmitters such as dopamine [39, 40] or serotonin [16, 17], both of which are known to influence behavioural changes during phase transition. Importantly, the insect gut microbiota should not be considered a static entity. Accumulating evidence indicates that both the composition and functional stability of insect gut microbial communities are dynamically modulated by a range of biotic and abiotic factors, including environmental stressors, pathogen exposure, dietary shifts, and changes in host physiological status [41, 42]. For example, recent studies have demonstrated that stress-related environmental perturbations can induce rapid and persistent alterations in gut microbial structure and function in insects [43, 44].

In the context of locusts, population density represents a major social and physical stressor, involving frequent tactile stimulation, altered feeding behaviour, and heightened metabolic demands. Such density-dependent stressors could plausibly first precipitate shifts in gut microbial communities, which may then be maintained over time and contribute to downstream changes in host neurophysiology and behaviour. This conceptual framework provides a mechanistic bridge linking environmental density, microbial divergence, and behavioural phase transition in locust grasshoppers, although this hypothesis has not yet been systematically tested.

Here, we analysed the taxonomic composition and metabolic potential of gut bacteria in *L. migratoria* to elucidate the relationship with host behavioural phase transition. We investigated gut bacterial compositions across the nymph-to-adult maturation stages in laboratory-reared solitary and gregarious individuals, and examined adult wild-type solitary individuals for comparison, using the 16S rRNA gene amplicon sequencing analysis. To gain further insight into microbial function, we also performed shotgun metagenomic sequencing analysis and identified microbial genes encoding neurotransmitter-metabolising enzymes. Our findings provide new insights into the potential microbial contributions to behavioural phase transitions in locusts.

## Materials and methods

### Sampling of the adult stage wild *L. migratoria* and their intestinal contents

Wild *L. migratoria* individuals were collected from two sites in Japan: the Kisegawa area in Shizuoka (35.102 N, 138.887E) and the Tamagawa area in Tokyo (35.650 N, 139.506E). All wild *L. migratoria* were identified as the solitary phase based on their body colour and distribution density. Three types of samples with temporal and spatial intervals were collected to identify the core gut microbes of *L. migratoria*: six individuals (Male:Female = 3:3) from Kisegawa in September 2022; nine (Male:Female = 5:4) from Kisegawa in July 2023; and nine (Male:Female = 4:5) from Tamagawa in September 2023. Each collected individual was immediately stored in a sterilised 50 ml centrifuge tube at −30°C. To capture field-derived data as accurately as possible, naturally excreted faeces and intestinal contents extracted by dissection were simultaneously collected.

### Laboratory-reared *L. migratoria*

To obtain the eggs of *L. migratoria,* wild individuals collected at Kisegawa area in September 2023 were reared in greenhouses at 30°C under a 12 h light / 12 h dark cycle and maintained at <60% relative humidity. All individuals were fed daily with fresh silver grass collected from a natural field at National Institute of Genetics (35.118 N, 138.937E). After egg hatching, first instar nymphs were moved under the distinct conditions to establish the solitary and gregarious individuals. For solitary individuals, each was reared in a 660 ml plastic container (isolated cage), while 200–300 individuals were reared in a 131 L cage (450 × 450 × 650 mm) to establish gregarious ones (high-density cage). The age of nymphs was determined according to the number of moults from the first to the fifth instar, and the duration of each nymphal stage was not considered, as it varied greatly among individuals even under identical conditions. To avoid horizontal propagation of microbes among the cages, isolated cages and high-density cages were incubated in the different greenhouses. All greenhouses were disinfected with ethanol every 72 h, and all breeding containers and breeding-related products were disinfected with ethanol every 24 h. To minimize the influence of laboratory adaptation, we annually established short-term laboratory lineages of *L. migratoria* by collecting wild individuals and rearing them for only two generations at our laboratory.

A modified behavioural analysis method, adapted from previously published methods [15, 16, 45] (see details in Supplementary Methods and Supplementary Fig. S1), was used to assess the behavioural phase distinction of laboratory-reared *L. migratoria* individuals. We defined the individuals reared in isolation as belonging to the solitary phase. In the 2023 season, 10 adult stage individuals were randomly selected from each of two groups: one reared at high population density for two generations, and the other reared in isolation (solitary group). The age of the adult individuals was not strictly standardized; however, behavioural analyses were performed within a consistent age range, between the third and tenth day after the final moult. Behavioural parameters were compared between groups using a *t*-test. Significant differences were observed in walking speed (*P* = .012), the fraction of time spent motionless (*P* = .021), and the total duration of movement (P = .021) (Supplementary Table S1). These results were further confirmed in the 2024 season, using a larger sample set of 48 individuals (24 per group: solitary vs gregarious), with all three behavioural parameters showing consistent and statically significant differences (walking speed: *P* < .001; time spent motionless: *P* = .0018; total duration of movement: *P* = .0016; Supplementary Table S2). Based on these reproducible results, we classified the high-density-reared group for two generations as a gregarious phase. The gregarious phase was applied not only to the adult stage but also to the nympal stages of the same generation. In this study, the term “gregarious” refers exclusively to groups that were reared under high population density for two consecutive generations, encompassing the entire life cycle of those individuals.

We additionally evaluated behavioural phase status using the *Pgreg* values, a logistic regression-based phase indicator developed in previous studies [15, 16, 45]. In our test, 67.6% of gregarious individuals and 82.4% of solitary individuals were correctly classified using *Pgreg*. Reflecting the characteristics of the binary logistic regression model [46, 47], this level of classification accuracy was considered acceptable.

### Faecal sample collection from laboratory-reared individuals

To minimize host DNA contamination, only faeces were collected from laboratory-reared *L. migratoria* individuals. For adult stages, each individual was transferred to an ethanol-disinfected isolated cage three days after adult emergence and kept there until it naturally excreted 400 mg of faeces. To assess the temporal stability of gut bacterial composition within individuals, an additional 400 mg of faeces was collected from the same individuals one week later using the same method. Accordingly, two sets of faecal samples were collected from adult individuals: on the third day and the 10th day after the final moult. Each faecal sample was collected in a 2 ml sterilized tube and stored in a −30°C refrigerator.

To investigate the developmental transition of gut bacterial composition, faeces were also collected from laboratory-reared nymphs at each of the five larval instar stages. Each solitary nymph was isolated in an ethanol-disinfected container for 2 h to obtain excreted faeces, then the nymph was returned to its original cage. This process was repeated every 24 h until obtaining 400 mg of faeces per individual. Due to the difficulty of tracking individuals in the high-density cages, we collected 800 mg of faeces excreted in the cages every week. All collected faeces were stored in a −30°C refrigerator.

### DNA isolation, quantification, and quality control

The QIAamp Fast DNA Stool Mini Kit (Qiagen GmbH, Hilden, Germany) was used for DNA extraction. The standard DNA isolation protocol from large volumes of stools was used for intestinal contents of wild individuals, while the modified protocol was used for the faecal samples from laboratory-reared individuals (see details in Supplementary Methods). Qubit (Life Technologies, CA, U.S.A.) was used for DNA quantification. The centrifugal concentration was used when the DNA quantity did not reach 1 ng/μl. The quality of the eluted DNA was checked with an Agilent 2100 Bioanalyzer (Agilent Technologies, CA, U.S.A.).

### 16S rRNA gene amplicon sequencing and bioinformatics analysis

The 515FY (5′-GTGYCAGCMGCCGCGGTAA) and 806RN (5′-GGACTACNVGGGTWTCTAAT) primer set of the 16S rRNA gene was used for PCR amplification [48]. The first PCR reaction mixture contained 1X KAPA HiFi HotStart ReadyMix (Kapa Biosystems, Wilmington, MA, USA), the primer set (0.2 μM each forward and reverse), and 1 ng of faecal DNA in a total volume of 25 μL per sample. PCR amplification conditions were as follows: an initial denaturation step at 95°C for 3 min, 25 denaturation cycles at 95°C for 30 s, annealing at 50°C for 30 s, an extension step at 72°C for 30 s, and a final extension at 72°C for 5 min. PCR products were purified using Agencourt AMPure XP (Beckman Coulter, Brea, CA, USA) and eluted in 50 μL of 10 mM Tris (pH 8.5) solution. The second PCR amplification was performed for eight cycles using 1X KAPA HiFi HotStart ReadyMix, 2.5 μL of each indexing PCR primer from the Nextera XT Index Kit (Illumina, San Diego, CA, USA) and 2.5 μL of purified PCR products in a 25 μL reaction mixture per sample. Libraries were sequenced on the Illumina NovaSeq 6000 platform and 150 bp paired-end reads were generated.

After obtaining the raw sequences, the DADA2 v.1.16 pipeline in R v.3.5 was used for taxonomic composition analysis [49]. Briefly, the “filterAndTrim” function was used for read quality filtering with the parameters “maxN=0, maxEE=c(2,2), truncQ=2, rm.phix=TRUE, trimLeft=c(19,20), truncLen=c(150,150)”. The “mergePairs” function was used to merge reverse and forward reads with the parameters “minOverlap = 7.” The reads were then deduplicated and ASVs (Amplicon Sequence Variants) were constructed. After read merging, the “removeBimeraDenovo” function was used to remove PCR chimera sequences. Taxonomy was assigned using the “assignTaxonomy” and “addSpecies” functions against the SILVA non-redundant small subunit rRNA gene sequence database version 138.1 [50]. All chloroplast and mitochondrial ASVs were removed. For ASVs that were not assigned a taxonomy, additional taxonomic classification was performed utilizing EZBioCloud [51].

### Shotgun metagenomic sequencing and bioinformatics analysis.

Shotgun metagenomic sequencing libraries of faecal DNA were generated using the Nextera FLEX kit (Illumina). The libraries were sequenced on the Illumina NovaSeq 6000 platform and 150 bp paired-end reads were generated. Adapter sequences and low-quality sequences were trimmed using fastp v.0.23.2 [52]. Then, host genomic reads were removed by mapping against the genome of *L. migratoria* (NCBI GenBank ID: GCA_026315105.1) by using BWA-MEM v.0.7.17 [53] with the default parameters. Quality-filtered read pairs were then assembled using MEGAHIT v.1.2.9 [54] with the default parameters. The taxonomic classification of contigs was inferred using Kraken2 v.2.0.8 [55] with its standard database and TaxonKit v.0.15 [56], and non-bacterial contigs were excluded. For contig binning and taxonomic abundance calculation, the read mapping result of BWA-MEM against the bacterial contigs was used. The protein-coding gene on the contigs was predicted using Prodigal v2.6.3 [57].

The function of each protein-coding gene on the contigs was inferred by DIAMOND v.2.0.15 [58] in sensitive mode against the KEGG protein sequence database [59] with sequence identity >40% and bit-score > 70. The read mapping result of BWA-MEM against the bacterial contigs was used to calculate the transcript per million (TPM) of each gene on the contigs [60], the KEGG Orthology (KO) composition was calculated to infer the difference in the composition of functional genes between the samples. To assess the biosynthetic capability of neurotransmitter compounds between samples, the compositions of 159 KOs, which were associated with metabolic pathways identified in the Gut-Brain Modules (GBMs) dataset [61], were examined in each sample. In addition, 13 KOs associated with a biosynthetic pathway of 4-vinylanisole, a key gregarious-phase pheromone in *L. migratoria*, and 12 KOs related to two biosynthetic pathways of pheylalanine, a potential biosynthetic precursor of 4-vinylanisole [62], were examined in each sample. GBMs and three biosynthetic pathways were defined as “identified” if all essential KOs constituting each pathway were simultaneously identified in a sample. Among the identified pathways, significant differences were assessed across all essential constituent KOs. A GBM or biosynthetic pathway was considered enriched in either solitary or gregarious individuals only when all of its essential KOs were significantly enriched in the same group. Metagenome-assembled genomes (MAGs) were constructed using MetaWRAP v.1.3.2 [63]. The taxonomic name of each MAG was inferred using GTDB-Tk v.2.1.1 [64]. Protein-coding genes in the MAGs were predicted and functionally annotated using DFAST v.1.2.0 [65].

### Statistical analysis

All statistical analyses were conducted using the R v.3.5. Beta diversities of taxonomic composition and gene functional composition were analysed using the vegan package [66]. Principal coordinate analysis (PCoA) was performed using a Bray–Curtis dissimilarity matrix based on ASV abundance to compare the beta diversity between groups. Permutational multivariate analysis of variance (PERMANOVA) was used to identify significant differences between the groups. The gene functional composition difference between the groups was analysed using principal component analysis (PCA). In addition, differential abundance analysis of KOs between solitary and gregarious individuals was performed using the limma package [67]. Only KOs with nonzero values in at least two samples within either group were included in the analysis. Statistical significance was determined based on an adjusted p-value (False Discovery Rate, Benjamini–Hochberg method) < 0.05 and an absolute log₂ fold change >1. Default parameters were used unless otherwise specified.

## Results

### Diversity and taxonomic composition of the gut microbiota of the adult wild *L. migratoria*

**Figure 1:**
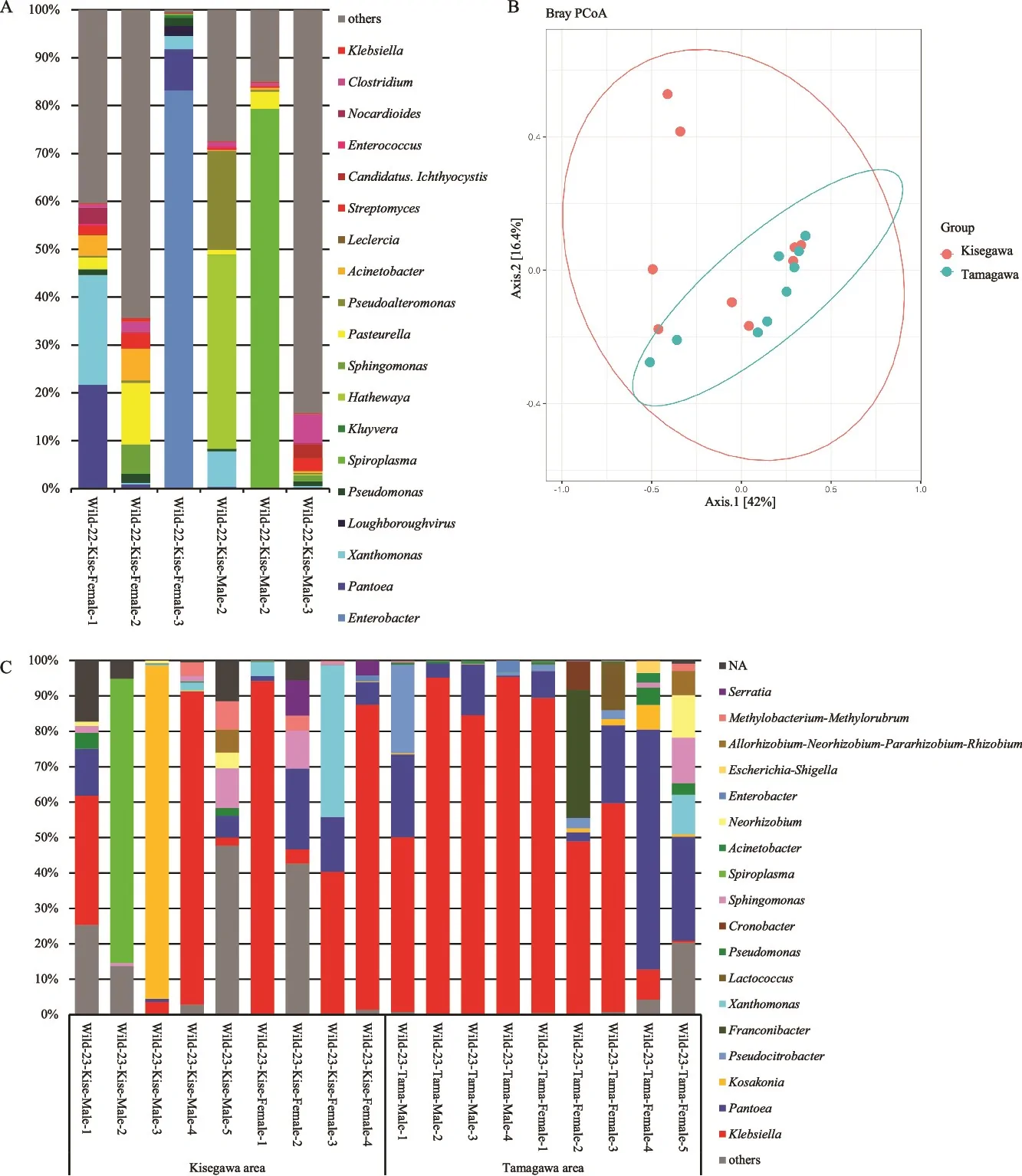
The diversity and taxonomic composition of the gut microbiota of wild-type *L. migratoria*. (A) the genus composition of the gut microbiota of wild-type *L. migratoria* using shotgun metagenomic sequencing. (B) PCoA plot of the 16S rRNA gene amplicon sequencing result showing the difference in gut microbial beta diversity between Kisegawa and Tamagawa areas. Each point represents a sample and each colour represents a sample group. 95% confidence ellipses are surrounded by oval circles. (C) the gut bacterial genus composition of wild-type *L. migratoria* collected from Kisewaga and Tamagawa areas.

An average of 67 M read pairs were obtained from the shotgun metagenomic sequencing of hindgut contents from six wild individuals (Supplementary Table S3). After quality filtering and removing host sequences, 4.5 M average paired reads remained. The genus-level taxonomic composition of the gut microbial communities was highly variable among samples; e.g. *Enterobacter* and *Spiroplasma* were specifically detected in a single female and male individuals, respectively (Fig. 1A and Supplementary Table S4). Notably, one sample (“Wild-22-Kise-Female-3”) contained an unusually high proportion of microbial DNA and a low proportion of host-derived DNA, which may have contributed to its distinct taxonomic profile. Such interindividual variation is not unexpected, as hindgut content samples likely include both epithelium-associated and luminal microbial populations that can differ in composition. Moreover, differences in the relative proportions of posterior and anterior hindgut regions included in each sample could also affect the observed community composition. Indeed, compositional differences between posterior and anterior hindgut regions have been reported in other insects such as *Odontotaenius disjunctus* [68]. The main purpose of this analysis was to provide an overview of the wild-type gut microbiota based on hindgut contents. Given the substantial interindividual variation observed, DNA extracted from faecal samples was used for subsequent analyses to obtain a more representative and comparable profile of the gut microbiota. Meanwhile, bacterial genera such as *Pseudomonas, Xanthomonas, Sphingomonas, Acinetobacter,* and *Klebsiella* were commonly detected in the most of samples.

Faecal samples from 18 wild-type individuals collected at two different locations (Kisegawa, July 2023, Female:Male = 4:5; Tamagawa, July 2023, Female:Male = 5:4) were analysed using 16S rRNA gene amplicon sequencing. We obtained a total of 71 K and 336 K quality-filtered read pairs from the Kisegawa and Tamagawa samples, respectively (Supplementary Table S5). PCoA (Fig. 1B) based on the Bray–Curtis dissimilarity indicates that Kisegawa samples have a slightly higher inter-sample variation, but no significant difference between the two locations (PERMANOVA: R^2^ = 0.07776, *P* = .18, method = Bray–Curtis dissimilarity). In these samples, *Klebsiella, Pantoea,* and *Sphingomonas* were commonly detected in 17 out of 18 samples, and *Pseudomonas* was observed in 16 out of 18 samples (Fig. 1C and Supplementary Table S5). As shown in Fig. 1A and Supplementary Table S4, *Klebsiella*, *Sphingomonas,* and *Pseudomonas* were also detected in all individuals analysed by shotgun metagenomic sequencing. Given that these bacterial genera were consistently observed across the individuals sampled at different locations and times, they are likely the candidates for core gut microbes of *L. migratoria*.

### Taxonomic compositional differences of the gut microbiota between laboratory-reared gregarious and solitary individuals

**Figure 2:**
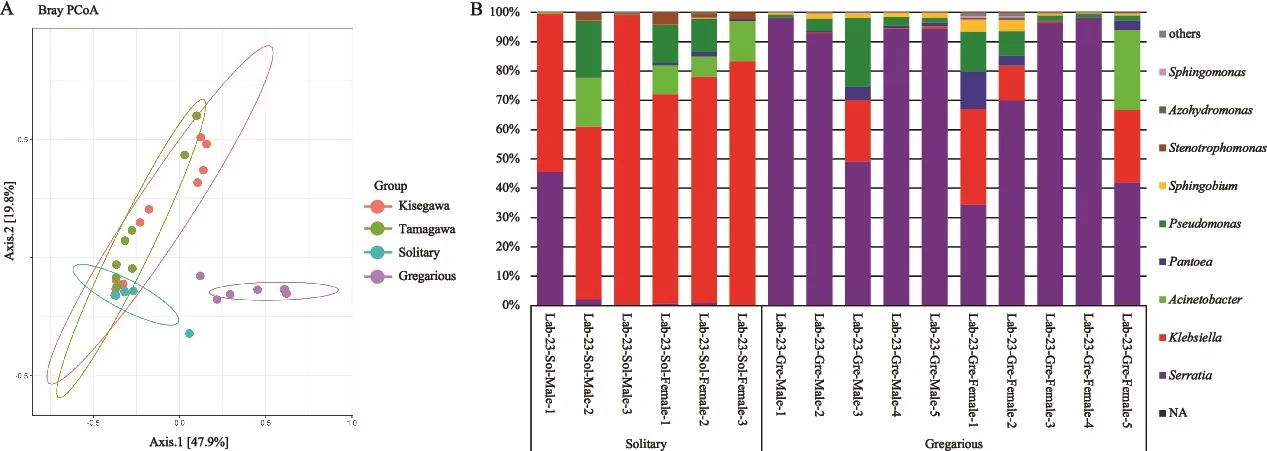
Compositional differences of the gut microbiota between gregarious and solitary individuals based on 16S rRNA gene amplicon sequencing. (A and B) the gut bacterial composition of laboratory-reared *L. migratoria* by 16S rRNA gene amplicon sequencing analysis. Taxonomic composition of the gut microbiota for 16 individuals of solitary (female:Male = 3:3) and gregarious (female:Male = 5:5) phase. (A) PCoA plot showing the difference in gut microbial beta diversity between solitary, gregarious, and wild (Tamagawa and Kisegawa) individuals. Each point represents a sample and each colour represents a sample group. 95% confidence ellipses are surrounded by oval circles. (B) the genus composition of the gut microbiota of laboratory-reared *L. migratoria*.

We performed 16S rRNA gene amplicon sequencing against 16 laboratory-reared individuals (Solitary: July 2023, Female:Male = 3:3; Gregarious: July 2023, Female:Male = 5:5). After quality filtering and chimera removal, we obtained an average of 39 K and 44 K paired reads from solitary and gregarious samples, respectively (Supplementary Table S5). Gut microbial genus composition significantly differed between laboratory-reared solitary and gregarious individuals (PERMANOVA: R^2^ = .70088, *P* = .001). PCoA (Fig. 2A) based on the Bray–Curtis dissimilarity showed that gregarious samples clustered separately from both wild and laboratory-reared solitary samples, whereas solitary samples overlapped with wild samples. These results indicate that gregarious individuals harbour a distinct gut microbial community not only from laboratory-reared solitary individuals but also from wild individuals.

In particular, *Serratia,* which was minor and less frequently observed in the wild and laboratory-reared solitary individuals (mean = 8.2%, SD = 18.3%), was enriched in the gregarious individuals (mean = 76.9%, SD = 25.8%). Conversely, *Klebsiella,* a candidate for core gut microbes of *L. migratoria*, was minor and even absent in the gregarious samples (mean = 9.2%, SD = 12.5%) compared to the solitary ones (mean = 73.93%, SD = 16.5%). Another candidate for core gut microbes, *Pseudomonas*, was not major in either the gregarious samples (mean = 6.0%, SD = 7.3%) or the solitary samples (mean = 7.3%, SD = 8.3%) (Fig. 2B and Supplementary Table S5). These results suggest that enrichment of *Serratia* and depletion of *Klebsiella* are associated with the phase transition of *L. migratoria*.

### Gene functional differences of the gut microbiome between gregarious and solitary individuals

**Figure 3:**
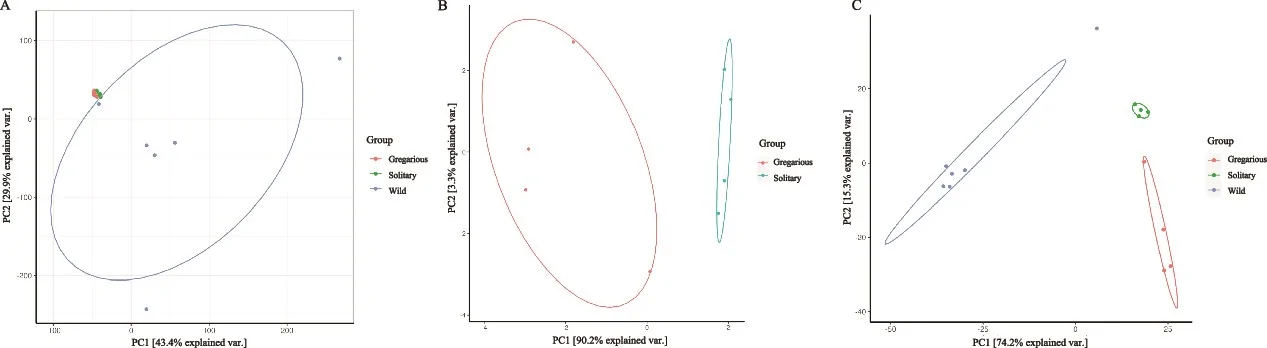
The gene functional composition of the gut microbiome of the reared *L. migratoria*. (A–C) PCA plot showing the gene functional composition of the gut microbiome of laboratory-reared *L. migratoria*. (A) PCA using all KOs of wild-collected or laboratory-reared individuals. (B) PCA using all KOs from solitary and gregarious individuals. (C) PCA using GBM-associated KOs from wild-collected and laboratory-reared individuals. Each point represents a sample and each colour represents a sample group. 95% confidence ellipses are surrounded by oval circles.

We conducted shotgun metagenomic sequencing analysis on faeces from four laboratory-reared solitary individuals and four gregarious individuals (Supplementary Table S3), yielding an average of 61.1 M and 60.1 M average paired reads, respectively. The age of all individuals was 10th days after the adult moulting. Genus composition was significantly different between groups (PERMANOVA: R^2^ = 0.99172, *P* = .023). The KEGG orthology composition analysis identified 3619 KOs from solitary samples and 3344 KOs from gregarious samples (Supplementary Table S6). Of these, 79 and 64 KOs were associated with GBM in solitary and gregarious samples, respectively (Supplementary Table S7). PCA using the composition of overall KOs (Fig. 3A and B) and the GBM-associated KOs (Fig. 3C) indicated that gregarious and solitary individuals have distinct functional gene compositions. PERMANOVA confirmed significant differences in gene functional composition between solitary and gregarious individuals using both total KOs (R^2^ = 0.73657, *P* = .023, method = Bray–Curtis dissimilarity) and GBM KOs (R^2^ = 0.73578, *P* = .024, method = Bray–Curtis dissimilarity).

The statistical comparison results of all KO compositions, including not only GBM-related but also other metabolic pathways, are presented in Supplementary Table S6. Using a limma-based statistical approach (adjusted *P* < .05, |log₂ fold change| > 1), we identified 257 KOs enriched in gregarious individuals and 374 KOs enriched in solitary individuals. Based on the DIAMOND annotations, KOs enriched in gregarious individuals were predominantly assigned to the genus *Serratia*, whereas those enriched in solitary individuals were primarily assigned to *Klebsiella* and *Pseudomonas*. The 257 KOs enriched in gregarious individuals included those related to the type II secretion system (K02452–K02465), urea metabolism (K11959–K11963, K01428–K01430, K01457, and K01941), and the conversion of tryptophan to kynurenine (K00453: tryptophan 2,3-dioxygenase and K01432: arylformamidase).

In contrast, the 374 KOs enriched in solitary individuals included multiple carbohydrate transporters (for glucose, mannose, xylose, and arabinose), nitrate/nitrite respiration (K00372, K02567, K02568, and K15576–K15578), vitamin B₁₂ biosynthesis (K00595, K02188, K02189, K02224–K02232, K03394, K06042, and K13541), tryptophan-to-indole-3-acetaldehyde conversion (K01593: aromatic-L-amino-acid/L-tryptophan decarboxylase and K00274: monoamine oxidase), GABA-to-succinate metabolism (K07250: 4-aminobutyrate aminotransferase, K08324: succinate-semialdehyde dehydrogenase, and K14268: 5-aminovalerate/4-aminobutyrate aminotransferase), and L-dopa-to-dopamine-to-homovanillic acid metabolism (K01593: aromatic-L-amino-acid/L-tryptophan decarboxylase and K00274: monoamine oxidase).

The solitary individuals showed higher abundances of genes related to GABA, indole, and dopamine metabolism (the latter two pathways share key enzymes), which may reflect a microbial potential for maintaining neurotransmitter homeostasis rather than promoting excitatory signaling. Although these interpretations remain speculative based on metagenomic data, the contrasting profiles may indicate differential microbial contributions to neurochemical balance between behavioural phases.

**Figure 4:**
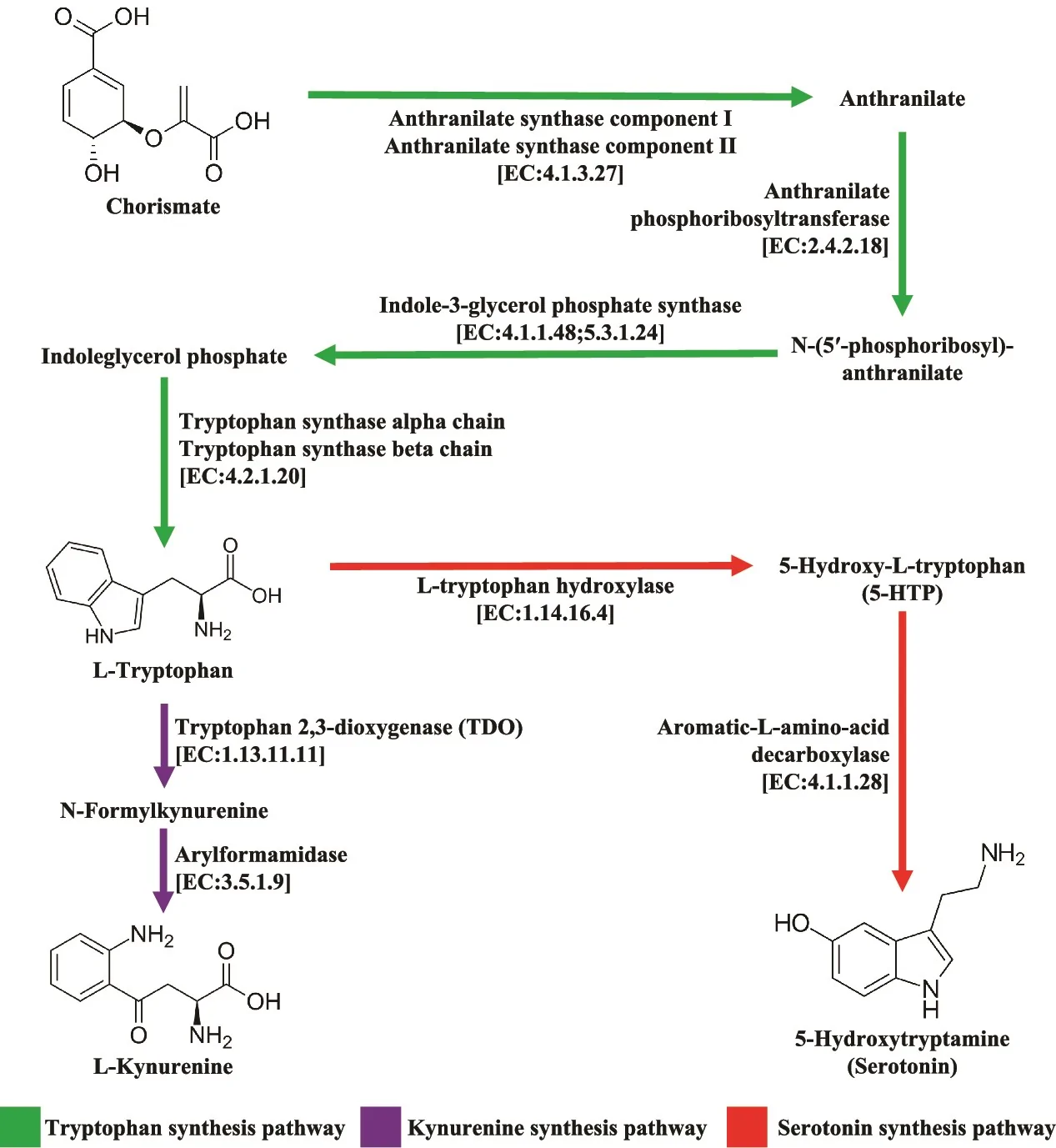
Profiles of the kynurenine and serotonin pathways in tryptophan metabolism.

Focusing on GBMs, nine GBM-related KOs (Supplementary Table S7) were enriched in solitary and gregarious samples, respectively. GBMs including these KOs were 10, and four of these GBMs—the kynurenine synthesis, the GABA degradation, the dopamine degradation, and the serotonin synthesis I—showed significant differences in their overall pathway abundance between gregarious and solitary individuals. Specifically, the overall pathway of the kynurenine synthesis pathway was enriched in gregarious samples. In the kynurenine synthesis pathway (Fig. 4), tryptophan is initially oxidized by tryptophan 2,3-dioxygenase (TDO) to yield N-formylkynurenine, which is subsequently hydrolyzed by arylformamidase to produce L-kynurenine. Both TDO (K00453: adjusted *P* = .009, |log₂ fold change| = 3.767) and arylformamidase (K01432: adjusted *P* = .007, |log₂ fold change| = 3.546) showed significantly higher TPM values in gregarious samples.

In contrast, the serotonin synthesis I, the GABA degradation, and the dopamine degradation pathways were enriched in solitary samples. In the serotonin synthesis I pathway, L-tryptophan is decarboxylated by L-tryptophan decarboxylase to produce tryptamine. In the dopamine degradation pathway, monoamine oxidase catalyzes the oxidative deamination of dopamine to 3,4-dihydroxyphenylacetaldehyde (DOPAL), accompanied by the production of ammonia and hydrogen peroxide as byproducts. In the GABA degradation pathway, 4-aminobutyrate aminotransferase (ABAT) catalyzes the conversion of gamma-aminobutyric acid (GABA) and 2-oxoglutarate into succinic semialdehyde and L-glutamate. All pathways comprises a single KO, and all enzymes—L-tryptophan decarboxylase (K01593: adjusted P = 0.043, |logary samples. In the se), monoamine oxidase (K00274: adjusted P = 0.042, |log0274: mples. In the se), and two 4-aminobutyrate aminotransferase (K07250: adjusted *P* = .009, |lognd two 4-aminobutyrate, K14268: *P* = .039, |log4268: *P* = .039, yrate)—showed significantly higher TPM values in solitary individuals.

These differences in GBM-associated genes suggest that variations in gut bacterial composition may contribute to behavioural phase transition in *L. migratoria*. Because GBM-related KOs correspond to processes involved in the synthesis or degradation of neuroactive compounds, these findings indicate the potential influence of gut microbes on host neurophysiology [61].

In parallel, three KOs associated with two 4-vinylanisole biosynthesis pathways and four KOs associated with the phenylalanine biosynthesis pathway showed significant differences between gregarious and solitary individuals (Supplementary Table S7). However, because some essential KOs required for the complete 4-vinylanisole biosynthesis pathway were not identified, the potential activity of this pathway originating from phenylalanine could not be inferred. In contrast, all essential KOs for phenylalanine biosynthesis were identified. Among them, chorismate mutase (K04093: adjusted *P* = .007, |log₂ fold change| = 3.545) showed significantly higher TPM values in gregarious individuals. Still, another KO with a similar function (K14187: adjusted *P* = .062, |log₂ fold change| = 0.228) did not differ significantly between the two groups. Consistently, no clear group-specific enrichment was observed in downstream steps, including cyclohexadienyl dehydratase (K01713: adjusted *P* = .034, |log₂ fold change| = 0.342) or in the final phenylalanine-producing step catalyzed by aromatic-amino-acid transaminase (K00832: adjusted *P* = .009, |log₂ fold change| = 0.586). Overall, despite some variation in individual KOs, no significant difference in the overall 4-vinylanisole biosynthesis pathway abundance was observed between gregarious and solitary individuals.

### Characteristics of metagenome-assembled genomes and annotated GBM-associated genes

A total of 34 MAGs belonging to 10 bacterial species were reconstructed from the shotgun metagenomic sequencing data with completeness >90% and contamination <5% (Table 1, Supplementary Table S8). Of these, eight MAGs were derived from wild samples, 19 from solitary samples, and seven from gregarious samples. Among them, *Serratia* and *Klebsiella*, which showed high association with gregarious and solitary samples respectively, were identified as *S. ureilytica* and *K. aerogenes*. No other species within these genera were detected. This suggests that the *Serratia* and *Klebsiella* found in the 16S rRNA gene amplicon and shotgun metagenomic sequences correspond to *S. ureilytica* and *K. aerogenes*.

**Table 1:** Summary of high-quality MAGs (completeness >90%, contamination <5%) showing the representative MAG with the highest completeness and lowest contamination for each species.

Code of the host	Type of the host	Species name	Contigs number	GC(%)	N50	Total contig length (bp)	CDS	Completeness (%)	Contamination (%)
Lab-23-Sol-Female-2	Gregarious	*Serratia ureilytica*	46	59.5	175 940	5,067,132	4702	97.21	1.068
Lab-23-Sol-Female-2	Gregarious	*Klebsiella aerogenes*	50	55.1	189 738	5,015,506	4633	97.99	1.006
Lab-23-Sol-Female-2	Gregarious	*Enterobacter hormaechei B*	49	55.7	164 223	4,438,642	4104	96.99	0.308
Lab-23-Sol-Male-3	Solitary	*Acinetobacter oleivorans*	32	38.5	230 827	3,929,983	3650	100	0.684
Lab-23-Sol-Female-1	Solitary	*Stenotrophomonas geniculata*	123	66.6	52 535	4,381,003	3989	98.03	0
Lab-23-Sol-Female-1	Gregarious	*Pantoea latae*	70	59.8	113 294	4,492,408	4100	90.53	0.402
Lab-23-Gre-Male-4	Gregarious	*Pantoea ananatis*	107	53.4	112 647	4,736,562	4389	93.03	1.315
Lab-23-Sol-Female-2	Solitary	*Pantoea endophytica*	60	54.8	130 066	4,687,411	4263	93.29	0
Wild-22-Kise-Female-3	Wild	*Xanthomonas albilineans*	96	63.1	71 461	3,502,053	2943	99.44	0.258
Wild-22-Kise-Male-2	Wild	*Spiroplasma phoeniceum*	236	26.6	6651	1,032,578	1875	93.58	0

Focusing on GBMs, a total of 79 GBM-related KOs and a total of 18 complete GBMs were identified from 10 representative MAGs (selected based on the highest completeness and lowest contamination for each taxon) (Table 2, Supplementary Table S9). Among the identified GBMs, the histamine synthesis pathway was found only in *K. aerogenes*, while the kynurenine degradation and the propionate degradation I pathways were detected only in *Stenotrophomonas geniculata*. The genes encoding the kynurenine synthesis pathway were found only in *S. ureilytica*, supporting the interpretation that this pathway is highly associated with gregarious individuals.

**Table 2:** Annotated GBMs and phenylalanine biosynthesis pathways in representative MAGs of 10 species. “Identified” indicates that all essential KOs of the metabolite pathway have been found.

Name of the metabolite pathway	Serratia ureilytica	Klebsiella aerogenes	Enterobacter hormaechei *B*	*Acinetobacter oleivorans*	*Stenotrophomonas geniculata*	*Pantoea latae*	Pantoea ananatis	*Pantoea endophytica*	Spiroplasma phoeniceum	Xanthomonas albilineans
Kynurenine synthesis [GBM004]	Identified									
Tryptophan synthesis [GBM005]	Identified	Identified	Identified	Identified	Identified		Identified			Identified
Glutamate synthesis I [GBM006]	Identified	Identified		Identified		Identified	Identified	Identified		
Histamine synthesis [GBM009]		Identified								
Dopamine degradation [GBM010]		Identified	Identified	Identified						
Kynurenine degradation [GBM018]					Identified					Identified
GABA degradation [GBM019]		Identified			Identified	Identified				Identified
GABA synthesis I [GBM020]	Identified	Identified				Identified				
GABA synthesis II [GBM021]		Identified	Identified							
Dopamine degradation [GBM023]		Identified	Identified	Identified						
Nitric oxide degradation I [GBM027]	Identified	Identified		Identified		Identified	Identified	Identified		Identified
ClpB (ATP-dependent chaperone protein) [GBM029]	Identified	Identified	Identified	Identified	Identified	Identified	Identified	Identified	Identified	Identified
Quinolinic acid degradation [GBM033]		Identified	Identified			Identified	Identified			
S-Adenosylmethionine (SAM) synthesis [GBM036]	Identified	Identified	Identified	Identified	Identified	Identified	Identified	Identified	Identified	Identified
Inositol degradation [GBM038]		Identified				Identified	Identified	Identified		
Acetate synthesis I [GBM043]	Identified		Identified			Identified	Identified	Identified		
Acetate degradation [GBM047]	Identified	Identified	Identified	Identified	Identified	Identified	Identified	Identified		Identified
Propionate degradation I [GBM056]					Identified					
Phenylalanine biosynthesis pathway [M00022]	Identified	Identified	Identified	Identified		Identified	Identified	Identified		

Since a high abundance of *Serratia* and a low abundance of *Klebsiella* is a common feature in the gut bacterial composition of gregarious samples, it could be estimated that increasing kynurenine synthesis and decreasing histamine synthesis are also associated with the gregarious phase transition. These observations suggest a differential effect of gut microbes on the host between the solitary and gregarious phases, as the associated genes were previously curated as GBMs [61], each corresponding to a specific neuroactive compound or degradation pathway. Based on these prior findings, a potential causal relationship between the gregarious phase transition and the activity of these two metabolic pathways might exist.

In parallel, the complete phenylalanine biosynthesis pathway was identified in all representative MAGs except for the *S. phoeniceum* MAG (Table 2, Supplementary Table S9). In contrast, no MAG contained a complete 4-vinylanisole biosynthesis pathway. These results suggest that phenylalanine could potentially be produced by members of the gut microbes of *L. migratoria*, whereas 4-vinylanisole is unlikely to be microbially derived. However, because the phenylalanine biosynthesis pathway was present across nine MAGs, including *S. ureilytica* and *K. aerogenes*, the potential for strain-specific differences in phenylalanine biosynthetic capacity is likely to be limited.

### A high abundance of *Serratia* can be maintained in the gregarious phase from the nymph to the adult stage.

**Figure 5:**
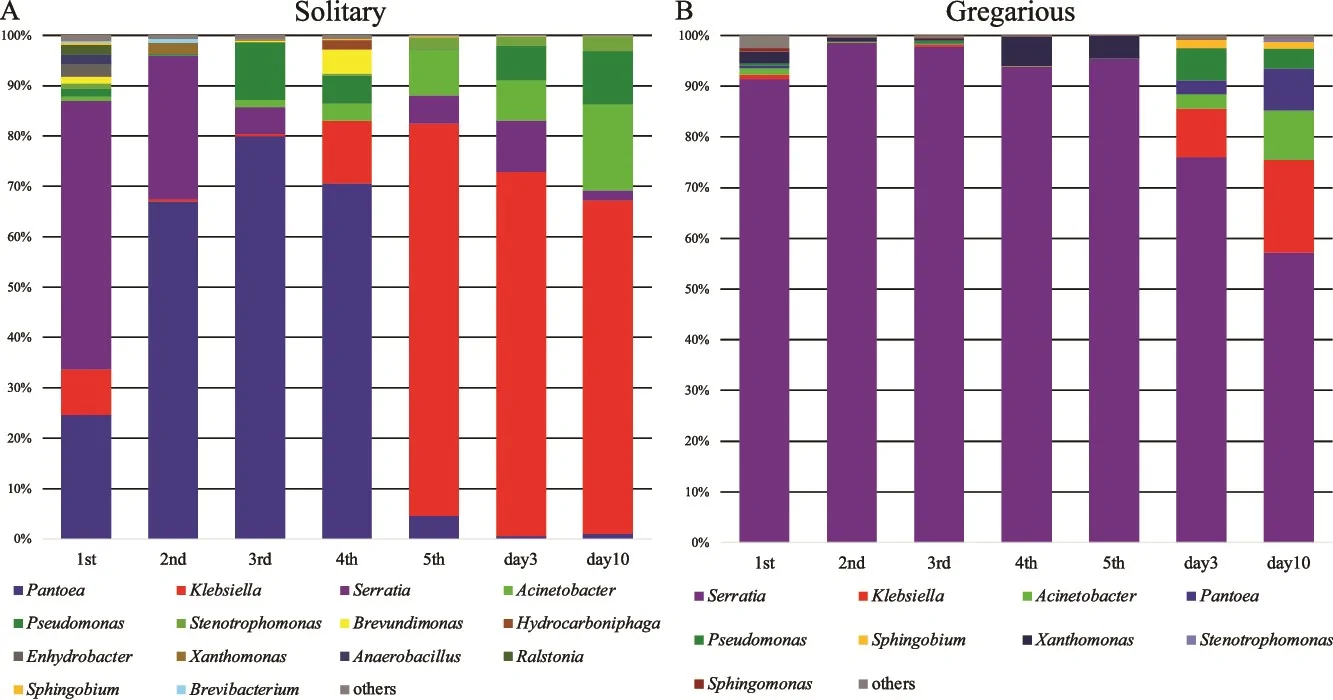
Transition of gut bacterial composition during the nymph (stages from first to fifth) to the adult (third day and 10th day) maturation step. The 16S rRNA gene amplicon sequencing analysis results of (A) the solitary group and (B) the gregarious group.

The gut bacterial composition based on 16S rRNA gene amplicon sequencing analysis was compared between gregarious (10 samples per an adult stage, two samples per a nymph stage) and solitary sample (six samples per a stage) during their growth. In solitary individuals, changes in the gut bacterial composition were observed within a short period (Fig. 5A and Supplementary Table S5). In the first nymph stage, the abundance of *Serratia* was highest at 54.27%, followed by *Pantoea* at 24.58%, and *Klebsiella* at 9.08%. This composition generally changed with growth, and at the fourth nymph stage, *Pantoea* became predominant (70.60%), and *Serratia* was no longer detected. After the fifth moulting, the composition changed dynamically, and on the 10th day of the adult stage, *Klebsiella* dominated (77.27%), while *Pantoea* (0.94%) and *Serratia* (2.03%) became minor taxa. On the other hand, in the gregarious individuals, the early gut bacterial composition was maintained until the adult stage (Fig. 5B and Supplementary Table S5). In particular, *Serratia* remained the dominant genus from the nymph to the adult stage, maintaining >90% relative abundance during the nymph stages. After the adult moulting, although the abundance of *Serratia* generally decreased, it remained dominant reaching 57.23% on the 10th day of the adult stage.

These results suggest that the gut bacterial composition changes more dynamically during maturation in the solitary phase individuals, but remains stable in the gregarious phase individuals. The persistent dominance of *Serratia* across the gregarious life stage implies a strong association with the gregarious phase. Given the identification of kynurenine synthesis genes and other metabolic genes in the MAG of *S. ureilyticia*, our finding suggests that functional traits of gut microbes may be consistently maintained throughout the life of the gregarious phase *L. migratoria*.

## Discussion

### *Klebsiella*, a potential core gut microbe of the solitary *L. migratoria*.

Unlike mammals, some insects lack core gut microbes [69], making it essential to assess whether specific microbial taxa are consistently associated with a given host species. In this study, *Klebsiella, Pantoea, and Pseudomonas* were identified across nearly all *L. migratoria* samples. In particular, *Klebsiella* was consistently abundant in both wild and laboratory-reared solitary individuals. Although *Panotea* was the most dominant taxon until the fourth nymph stage, *Klebsiella* became predominant from the 5th stage onward. *Klebsiella* species, including *K. aerogenes*, have previously been reported to colonize the intestinal environment, either in mammals [70, 71] or in insects [72, 73]. In addition, a study reported that *K. aerogenes* has antifungal capabilities and shows a symbiotic relationship with *L. migratoria* [74]. These finding strongly suggest a that *Klebsiella* is a potential core gut microbe of *L. migratoria*.

We identified 53 KOs related to GBMs and 13 complete GBMs in the MAG of *K. aerogenes*, implying a potential role for this taxon in modulating host physiology. In particular, the histamine synthesis pathway was only identified in *K. aerogenes* among all reconstructed MAGs. Previous studies have shown that *K. aerogenes*-derived histamine can have a significant impact on mammalian abdominal pain [75]. In addition, studies in *Drosophila* have demonstrated that histamine plays key roles in various ecological and physiological processes in insects [76], including visual signal transmission [77], regulation of the sleep–wake cycle [78], courtship behaviour [79], and temperature sensing [80]. These observations raise the possibility of a gut-brain-microbiota axis in *L. migratoria* involving *K. aerogenes*. However, it is worth noting that *K. aerogenes* has also been reported as a pathogenic bacterium in honeybees [81]. Therefore, further experimental studies are needed to elucidate the detailed relationship between *L. migratoria* and *K. aerogenes*.

### Microbial differences between solitary and gregarious phases

We observed significant differences in gut bacterial composition between gregarious and solitary *L. migratoria*. While the abundance of *Klebsiella* was significantly depleted in gregarious individuals, *Serratia* was consistently dominant across all gregarious individuals regardless of maturation stage. Although a previous study reported the existence of *Serratia* in the solitary *L. migratoria* hindgut [38], *Serratia* was detected only in the first stage of nymphs and eggs. Considering these findings along with our observation, if *Serratia* encodes gene functions that affect the host, such effects may persist throughout the lifespan of gregarious *L. migratoria*.

A significant gene functional composition difference of the gut microbiome was observed between gregarious and solitary *L. migratoria* individuals. We identified 257 KOs enriched in gregarious individuals and 374 KOs enriched in solitary individuals. Among these, five and 10 KOs were related with GBMs in solitary and gregarious samples, respectively. In particular, the kynurenine synthesis genes were correlated with the gregarious phase. The kynurenine synthesis function is a pathway to synthesise L-kynurenine, an intermediate product such as kynurenic acid, xanthurenic acid, and nicotinic acid, from tryptophan. Since the kynurenine synthesis gene was annotated only in *S. ureilyticia* MAG, this gene was presumed to be derived from *Serratia*.

Several mammalian gut microbiome studies have shown that the kynurenine synthesis gene is functionally analogous to indoleamine 2,3-dioxygenase (IDO) [82, 83]. IDO-mediated pathways promote tryptophan degradation (Fig. 4), resulting in reduced serotonin synthesis [84–87]. The correlation between changes in serotonin concentration and the solitariness of the gregarious phase of *L. migratoria* has been reported previously [17]. Although direct evidence from insect gut microbiota demonstrating microbial TDO/IDO-mediated suppression of host serotonin synthesis is currently lacking, studies in social insects (e.g. honey bees) have shown that gut bacteria can modulate host tryptophan metabolism and reduce host TDO expression [29]. In our metagenomic data, the enrichment of microbial genes annotated as TDO/IDO suggests a potential microbial contribution to the tryptophan–kynurenine axis in *L. migratoria*. However, we emphasise that this remains a working hypothesis that requires further functional validation.

In parallel, pathways related to GABA, indole, and dopamine metabolism exhibited decreased TPM values in gregarious individuals. Notably, monoamine oxidase (MAO) that catalyzes the oxidative deamination of monoamine neurotransmitters such as serotonin and dopamine [88–90], showed a marked decrease in TPM values. Aromatic L-amino acid decarboxylase (AADC), which catalyzes several key decarboxylation reactions including the conversion of L-DOPA to dopamine and 5-hydroxytryptophan (5-HTP) to serotonin [91–93], showed a similar trend. This reduction in GBM-associated genes is likely attributable to the significant increase of *Serratia* in the gregarious group, as this taxon did not encode the corresponding functions. Previous studies have revealed that the dopamine and the serotonin pathways have a crucial role in determining the behavioural phase of *L. migratoria* [17, 39, 40]. These findings suggest that MAO may be associated in the gregarious phase transition by affecting the dopamine and serotonin levels in *L. migratoria*.

We also identified a complete phenylalanine biosynthesis pathway in the gut microbiome. Phenylalanine serves as the initial substrate for the biosynthesis of 4-vinyanisole, a key gregarious-phase pheromone in *L. migratoria* [62]. Although a previous study reported that this substrate is primarily derived from plant sources [62], our findings suggest that gut microbiota-derived phenylalanine may also contribute to 4-vinylanisole production. However, the phenylalanine biosynthesis pathway was present in both *S. ureilytica* and *K. aerogenes*, and no significant difference in its abundance was observed between gregarious and solitary individuals. In addition, 4-vinylanisole biosynthesis pathways were not identified in our observation, which indicates that 4-vinylanisole itself is not synthesized in gut microbes. Therefore, the contribution of microbially synthesised phenylalanine to 4-vinylanisole production is likely limited under the conditions examined in this study.

This study has some limitations. First, because the tryptophan synthesis pathway was also identified in *S. ureilytica*, it remains unclear whether kynurenine synthesis plays an important role in regulating tryptophan and serotonin levels in the *L. migratoria* gut. In addition, there is currently no direct evidence that MAO and AADC derived from gut microbes can affect dopamine levels in the host brain. Furthermore, the prediction of microbial metabolic capabilities in this study relied on datasets constructed primarily from mammalian research, such as the GBM dataset. While bacterial genes themselves do not vary depending on the host, mammals and insects diverged ~700 million years ago [94], and their gut environments differ markedly in temperature, structure, and digestive system [95, 96]. Given that several studies have reported that environmental factors can strongly influence bacterial gene expression [97–100], it remains unclear whether the gene functions identified here are actually expressed in the gut environment of *L. migratoria*. Another important limitation concerns microbial quantification. While metagenomic sequencing enabled the characterization of gut microbial composition and relative abundance, absolute quantification of bacterial taxa and functional genes was not performed in this study. As a result, changes in relative abundance cannot be unequivocally distinguished from variations in total microbial load between solitary and gregarious phases. Absolute quantification approaches, such as quantitative PCR (qPCR) targeting universal or taxon-specific 16S rRNA genes, would allow more precise estimation of microbial population sizes and functional gene copy numbers, thereby enabling a more accurate interpretation of microbial contributions to host physiology. Due to sample limitations, such analyses could not be conducted in the present study. Nevertheless, future studies integrating metagenomic profiling with absolute quantification methods including qPCR and spike-in standards will be essential to disentangle relative versus absolute microbial changes and to clarify their functional relevance. Together, these limitations underscore the need for further experimental and quantitative investigations to establish the functional significance and causal mechanisms underlying microbe-mediated behavioural phase transitions in locusts.

## Conclusion

In this study, we identified a strong correlation between the gut bacterial composition and the gregarious phase transition of *L. migratoria.* Gregarious individuals exhibited a consistently high abundance of *Serratia* and reduction of other taxa, including *Klebsiella*, compared to solitary individuals. This compositional differences were maintained throughout the life of the gregarious phase *L. migratoria*. The gene function composition analysis also showed significant differences between gregarious and solitary individuals. In particular, gregarious individuals were enriched in kynurenine biosynthesis genes, while solitary individuals enriched GABA, indole, and dopamine metabolism genes. These results support the hypothesis that microbial community structure and function may contribute to the gregarious phase transition of *L. migratoria*.

## Supplementary Material

Figure_S1_revision_ycag009

Figure_S2_revision_ycag009

Supplementary_Table_S1_revision_ycag009

Supplementary_Table_S2_revision_ycag009

Supplementary_Table_S3_revision_ycag009

Supplementary_Table_S4_revision_ycag009

Supplementary_Table_S5_revision_ycag009

Supplementary_Table_S6_revision_ycag009

Supplementary_Table_S7_revision_ycag009

Supplementary_Table_S8_revision_ycag009

Supplementary_Table_S9_revision_ycag009

supplementary_material_revision_20251231_ycag009

## Data Availability

The 16S rRNA gene amplicon sequencing data and the shotgun metagenomic sequencing data have been registered in the DDBJ under the BioProject numbers PRJDB20285 and PRJDB20286. The metagenome assembled genome sequence data of *S. ureilytica* is available in the DDBJ under the accession number BAABXD010000001-BAABXD010000091. The BioProject number is PRJDB20287. The megagenomic sequencing raw read is deposited under DRA021614 with the BioSample accession numbers SAMD01593440 and SAMD01593441.

## References

[1] Herbillon F, Piou C, Meynard CN. An increase in management actions has compensated for past climate change effects on desert locust gregarization in western Africa. *Heliyon.* 2024;10:e29231. 10.1016/j.heliyon.2024.e29231 38644897 PMC11033115

[2] Zhang L, Lecoq M, Latchininsky A. et al. Locust and grasshopper management. *Annu Rev Entomol* 2019;64:15–34. 10.1146/annurev-ento-011118-112500 30256665

[3] Marion LG, Rick O, Arianne C. A global review on locusts (Orthoptera: Acrididae) and their interactions with livestock grazing practices. *Front Ecol Evol* 2019;7:263. 10.3389/fevo.2019.00263

[4] Peng W, Ma NL, Zhang D. et al. A review of historical and recent locust outbreaks: links to global warming, food security and mitigation strategies. *Environ Res* 2020;191:110046. 10.1016/j.envres.2020.110046 32841638

[5] Chapuis MP, Loiseau A, Michalakis Y. et al. Outbreaks, gene flow and effective population size in the migratory locust, locusta migratoria: a regional-scale comparative survey. *Mol Ecol* 2009;18:792–800. 10.1111/j.1365-294X.2008.04072.x 19207256

[6] Latchininsky A, Sword G, Sergeev M. et al. Locusts and grasshoppers: behavior, ecology, and biogeography. *Psyche: A Journal of Entomology* 2011;578327. 10.1155/2011/578327

[7] Song H . Density-dependent phase Polyphenism in nonmodel locusts: a minireview. *Psyche: A Journal of Entomology* 2021;741769. 10.1155/2011/741769

[8] Bazazi S, Buhl C, Hale JJ. et al. Collective motion and cannibalism in locust migratory bands. *Curr Biol* 2008;18:735–9. 10.1016/j.cub.2008.04.035 18472424

[9] Couzin ID, Couzin-Fuchs E. The chemical ecology of locust cannibalism. *Science.* 2023;380:454–5. 10.1126/science.adh5264 37141343

[10] Guttal V, Romanczuk P, Simpson SJ. et al. Cannibalism can drive the evolution of behavioural phase polyphenism in locusts. *Ecol Lett* 2012;15:1158–66. 10.1111/j.1461-0248.2012.01840.x 22882379

[11] Chang H, Cassau S, Krieger J. et al. A chemical defense deters cannibalism in migratory locusts. *Science.* 2023;380:537–43. 10.1126/science.ade6155 37141362

[12] Sword GA, Simpson SJ, El Hadi OT. et al. Density-dependent aposematism in the desert locust. *Proc Biol Sci* 2000;267:63–8. 10.1098/rspb.2000.0967 10670954 PMC1690497

[13] Yamagishi M, Tanaka S. Overwintering biology and morphological characteristics of the migratory locust, *locusta migratoria* after outbreaks on Iheya Island. *Japan Applied Entomology and Zoology* 2009;44:165–74. 10.1303/aez.2009.165

[14] Guo X, Kang L. Phenotypic plasticity in locusts: trade-off between migration and reproduction. *Annu Rev Entomol* 2025;70:23–44. 10.1146/annurev-ento-013124-124333 39227131

[15] Simpson SJ, Despland E, Hägele BF. et al. Gregarious behavior in desert locusts is evoked by touching their back legs. *Proc Natl Acad Sci USA* 2001;98:3895–7. 10.1073/pnas.071527998 11274411 PMC31149

[16] Anstey ML, Rogers SM, Ott SR. et al. Serotonin mediates behavioral gregarization underlying swarm formation in desert locusts. *Science.* 2009;323:627–30. 10.1126/science.1165939 19179529

[17] Guo X, Ma Z, Kang L. Serotonin enhances solitariness in phase transition of the migratory locust. *Front Behav Neurosci* 2013;7:129. 10.3389/fnbeh.2013.00129 24109441 PMC3791384

[18] Song H, Mariño-Pérez RG, Woller DA. et al. Evolution, diversification, and biogeography of grasshoppers (Orthoptera: Acrididae). *Insect Systematics and Diversity* 2018;2:3. 10.1093/isd/ixy008

[19] Dillon RJ, Vennard CT, Charnley AK. A note: gut bacteria produce components of a locust cohesion pheromone. *J Appl Microbiol* 2002;92:759–63. 10.1046/j.1365-2672.2002.01581.x 11966918

[20] Lavy O, Gophna U, Gefen E. et al. The effect of density-dependent phase on the locust gut bacterial composition. *Front Microbiol* 2019;9:3020. 10.3389/fmicb.2018.03020 30713526 PMC6345702

[21] Lavy O, Lewin-Epstein O, Bendett Y. et al. Microbiome-related aspects of locust density-dependent phase transition. *Environ Microbiol* 2022;24:507–16. 10.1111/1462-2920.15883 35068041

[22] Shi W, Guo Y, Xu C. et al. Unveiling the mechanism by which microsporidian parasites prevent locust swarm behavior. *Proc Natl Acad Sci USA* 2014;111:1343–8. 10.1073/pnas.1314009111 24474758 PMC3910566

[23] Tan SQ, Zhang KQ, Chen HX. et al. The mechanism for microsporidian parasite suppression of the hindgut bacteria of the migratory locust *locusta migratoria manilensis*. *Sci Rep* 2015;5:17365. 10.1038/srep17365 26612678 PMC4661595

[24] Ahmed H, Leyrolle Q, Koistinen V. et al. Microbiota-derived metabolites as drivers of gut-brain communication. *Gut Microbes* 2022;14:2102878. 10.1080/19490976.2022.2102878 35903003 PMC9341364

[25] Fischer CN, Trautman EP, Crawford JM. et al. Metabolite exchange between microbiome members produces compounds that influence *drosophila* behavior. *Elife.* 2017;6:e18855. 10.7554/eLife.18855 28068220 PMC5222558

[26] Jia Y, Jin S, Hu K. et al. Gut microbiome modulates drosophila aggression through octopamine signaling. *Nat Commun* 2021;12:2698. 10.1038/s41467-021-23041-y 33976215 PMC8113466

[27] Fioriti F, Rifflet A, Gomperts Boneca I. et al. Bacterial peptidoglycan serves as a critical modulator of the gut-immune-brain axis in drosophila. *Brain Behav Immun* 2024;119:878–97. 10.1016/j.bbi.2024.05.009 38710338

[28] Liberti J, Kay T, Quinn A. et al. The gut microbiota affects the social network of honeybees. *Nat Ecol Evol* 2022;6:1471–9. 10.1038/s41559-022-01840-w 35995848 PMC7613669

[29] Zhang Z, Mu X, Cao Q. et al. Honeybee gut lactobacillus modulates host learning and memory behaviors via regulating tryptophan metabolism. *Nat Commun* 2022;13:2037. 10.1038/s41467-022-29760-0 35440638 PMC9018956

[30] Zheng H, Powell JE, Steele MI. et al. Honeybee gut microbiota promotes host weight gain via bacterial metabolism and hormonal signaling. *Proc Natl Acad Sci USA* 2017;114:4775–80. 10.1073/pnas.1701819114 28420790 PMC5422775

[31] Liberti J, Engel P. The gut microbiota - brain axis of insects. *Curr Opin Insect Sci* 2020;39:6–13. 10.1016/j.cois.2020.01.004 32078985

[32] Chabanol E, Gendrin M. Insects and microbes: best friends from the nursery. *Curr Opin*. *Insect Sci* 2024;66:101270. 10.1016/j.cois.2024.101270 39293738

[33] Brune A, Dietrich C. The gut microbiota of termites: digesting the diversity in the light of ecology and evolution. *Ann Rev Microbiol* 2015;69:145–66. 10.1146/annurev-micro-092412-155715 26195303

[34] Matsuura K . Nestmate recognition mediated by intestinal bacteria in a termite. *Reticulitermes speratus Oikos* 2001;92:20–6. 10.1034/j.1600-0706.2001.920103.x

[35] Mevers E, Chouvenc T, Su NY. et al. Chemical interaction among termite-associated microbes. *J Chem Ecol* 2017;43:1078–85. 10.1007/s10886-017-0900-6 29134406 PMC5735195

[36] Teseo S, Van Zweden JS, Pontieri L. et al. The scent of symbiosis: gut bacteria may affect social interactions in leaf-cutting ants. *Anim Behav* 2019;150:239–54. 10.1016/j.anbehav.2018.12.017

[37] Griffiths JA, Nirmalkar K, Wu WL. et al. The gut microbiome shapes social behaviour across animal species. *Nat Rev Microbiol in press* 2025. 10.1038/s41579-025-01262-y 41238755

[38] Li K, Li W-J, Liang K. et al. Gut microorganisms of *locusta migratoria* in various life stages and its possible influence on cellulose digestibility. *mSystems.* 2024;9:e0060024. 10.1128/msystems.00600-24 38888356 PMC11264664

[39] Ma Z, Guo W, Guo X. et al. Modulation of behavioral phase changes of the migratory locust by the catecholamine metabolic pathway. *Proc Natl Acad Sci USA* 2011;108:3882–7. 10.1073/pnas.1015098108 21325054 PMC3053982

[40] Yang M, Wei Y, Jiang F. et al. MicroRNA-133 inhibits behavioral aggregation by controlling dopamine synthesis in locusts. *PLoS Genet* 2014;10:e1004206. 10.1371/journal.pgen.1004206 24586212 PMC3937255

[41] Yasiki Y, Shivakumar MS. A comprehensive account of functional role of insect gut microbiome in insect orders. *Journal of Natural Pesticide Research* 2024;11:100110. 10.1016/j.napere.2024.100110

[42] Wang Y, Tang J, Chen Y. et al. The ecological-evolutionary game of the insect gut microbiome: environmental drivers, host regulation, and prospects for cross-cutting applications. *Vet Sci* 2025;12:866. 10.3390/vetsci12090866 41012791 PMC12474139

[43] Zhang Y, Xu H, Tu C. et al. Enhanced capacity of a leaf beetle to combat dual stress from entomopathogens and herbicides mediated by associated microbiota. *Integr Zool* 2024;19:1092–104. 10.1111/1749-4877.12812 38379126

[44] Hafsi A, Moquet L, Hendrycks W. et al. Evidence for a gut microbial community conferring adaptability to diet quality and temperature stressors in phytophagous insects: the melon fruit fly *Zeugodacus cucurbitae* (Diptera: Tephritidae) as a case study. *BMC Microbiol* 2024;24:514. 10.1186/s12866-024-03673-y 39627693 PMC11613556

[45] Guo W, Wang X, Ma Z. et al. CSP and takeout genes modulate the switch between attraction and repulsion during behavioral phase change in the migratory locust. *PLoS Genet* 2011;7:e1001291. 10.1371/journal.pgen.1001291 21304893 PMC3033386

[46] Bujang MA, Sa'at N, Sidik TMITAB. et al. Sample size guidelines for logistic regression from observational studies with large population: emphasis on the accuracy between statistics and parameters based on real life clinical data. *Malays J Med Sci* 2018;25:122–30. 10.21315/mjms2018.25.4.12 30914854 PMC6422534

[47] Ranganathan P, Pramesh CS, Aggarwal R. Common pitfalls in statistical analysis: logistic regression. *Perspect Clin Res* 2017;8:148–51. 10.4103/picr.PICR_87_17 28828311 PMC5543767

[48] Mori H, Kato T, Ozawa H. et al. Assessment of metagenomic workflows using a newly constructed human gut microbiome mock community. *DNA Res* 2023;30:dsad010. 10.1093/dnares/dsad010 37253538 PMC10229288

[49] Callahan BJ, McMurdie PJ, Rosen MJ. et al. DADA2: high-resolution sample inference from Illumina amplicon data. *Nat Methods* 2016;13:581–3. 10.1038/nmeth.3869 27214047 PMC4927377

[50] Quast C, Pruesse E, Yilmaz P. et al. The SILVA ribosomal RNA gene database project: improved data processing and web-based tools. *Nucleic Acids Res* 2013;41:D590–6. 10.1093/nar/gks1219 23193283 PMC3531112

[51] Yoon SH, Ha SM, Kwon S. et al. Introducing EzBioCloud: a taxonomically united database of 16S rRNA gene sequences and whole-genome assemblies. *Int J Syst Evol Microbiol* 2017;67:1613–7. 10.1099/ijsem.0.001755 28005526 PMC5563544

[52] Chen S, Zhou Y, Chen Y. et al. Fastp: an ultra-fast all-in-one FASTQ preprocessor. *Bioinformatics.* 2018;34:i884–90. 10.1093/bioinformatics/bty560 30423086 PMC6129281

[53] Li H, Durbin R. Fast and accurate short read alignment with burrows-wheeler transform. *Bioinformatics.* 2009;25:1754–60. 10.1093/bioinformatics/btp324 19451168 PMC2705234

[54] Li D, Liu CM, Luo R. et al. MEGAHIT: an ultra-fast single-node solution for large and complex metagenomics assembly via succinct de Bruijn graph. *Bioinformatics.* 2015;31:1674–6. 10.1093/bioinformatics/btv033 25609793

[55] Wood DE, Lu J, Langmead B. Improved metagenomic analysis with kraken 2. *Genome Biol* 2019;20:257. 10.1186/s13059-019-1891-0 31779668 PMC6883579

[56] Shen W, Ren H. TaxonKit: a practical and efficient NCBI taxonomy toolkit. *J Genet Genomics* 2021;48:844–50. 10.1016/j.jgg.2021.03.006 34001434

[57] Hyatt D, Chen GL, Locascio PF. et al. Prodigal: prokaryotic gene recognition and translation initiation site identification. *BMC Bioinformatics* 2010;11:119. 10.1186/1471-2105-11-119 20211023 PMC2848648

[58] Buchfink B, Reuter K, Drost HG. Sensitive protein alignments at tree-of-life scale using DIAMOND. *Nat Methods* 2021;18:366–8. 10.1038/s41592-021-01101-x 33828273 PMC8026399

[59] Kanehisa M, Sato Y, Kawashima M. et al. KEGG as a reference resource for gene and protein annotation. *Nucleic Acids Res* 2016;44:D457–62. 10.1093/nar/gkv1070 26476454 PMC4702792

[60] Li B, Ruotti V, Stewart RM. et al. RNA-Seq gene expression estimation with read mapping uncertainty. *Bioinformatics.* 2010;26:493–500. 10.1093/bioinformatics/btp692 20022975 PMC2820677

[61] Valles-Colomer M, Falony G, Darzi Y. et al. The neuroactive potential of the human gut microbiota in quality of life and depression. *Nat Microbiol* 2019;4:623–32. 10.1038/s41564-018-0337-x 30718848

[62] Guo X, Gao L, Li S. et al. Decoding 4-vinylanisole biosynthesis and pivotal enzymes in locusts. *Nature.* 2025;644:420–9. 10.1038/s41586-025-09110-y 40562929 PMC12350148

[63] Uritskiy GV, DiRuggiero J, Taylor J. MetaWRAP-a flexible pipeline for genome-resolved metagenomic data analysis. *Microbiome.* 2018;6:158. 10.1186/s40168-018-0541-1 30219103 PMC6138922

[64] Chaumeil PA, Mussig AJ, Hugenholtz P. et al. GTDB-Tk: a toolkit to classify genomes with the genome taxonomy database. *Bioinformatics.* 2019;36:1925–7. 10.1093/bioinformatics/btz848 31730192 PMC7703759

[65] Tanizawa Y, Fujisawa T, Nakamura Y. DFAST: a flexible prokaryotic genome annotation pipeline for faster genome publication. *Bioinformatics.* 2018;34:1037–9. 10.1093/bioinformatics/btx713 29106469 PMC5860143

[66] Dixon P . VEGAN, a package of R functions for community ecology. *J Veg Sci* 2003;14:927–30. 10.1111/j.1654-1103.2003.tb02228.x

[67] Ritchie ME, Phipson B, Wu D. et al. Limma powers differential expression analyses for RNA-sequencing and microarray studies. *Nucleic Acids Res* 2015;43:e47. 10.1093/nar/gkv007 25605792 PMC4402510

[68] Ceja-Navarro JA, Nguyen NH, Karaoz U. et al. Compartmentalized microbial composition, oxygen gradients and nitrogen fixation in the gut of *Odontotaenius disjunctus*. *ISME J* 2014;8:6–18. 10.1038/ismej.2013.134 23985746 PMC3869013

[69] Hammer TJ, Janzen DH, Hallwachs W. et al. Caterpillars lack a resident gut microbiome. *Proc Natl Acad Sci USA* 2017;114:9641–6. 10.1073/pnas.1707186114 28830993 PMC5594680

[70] Merciecca T, Bornes S, Nakusi L. et al. Role of *Klebsiella pneumoniae* type VI secretion system (T6SS) in long-term gastrointestinal colonization. *Sci Rep* 2022;12:16968. 10.1038/s41598-022-21396-w 36216848 PMC9550808

[71] Wang X, Meng M, Sun J. et al. *Klebsiella aerogenes* exacerbates colon tumorigenesis in the AOM/DSS-induced C57BL/6J mouse. *Biochem Biophys Res Commun* 2024;694:149410. 10.1016/j.bbrc.2023.149410 38134478

[72] Yin Y, Wang S, Zhang K. et al. *Klebsiella pneumoniae* in the intestines of *Musca domestica* larvae can assist the host in antagonizing the poisoning of the heavy metal copper. *BMC Microbiol* 2023;23:383. 10.1186/s12866-023-03082-7 38049761 PMC10694927

[73] Abdurehman DA, Abdurahman MK. Isolation, assessments of risk factors, and antimicrobial susceptibility test of *Klebsiella* from gut of bee in and around Haramaya University bee farm, east Hararghe, Oromia regional state, Ethiopia. *Vet Med Int* 2022;2022:9460543. 10.1155/2022/9460543 35942202 PMC9356775

[74] Tan S, Wei H, Camara I. et al. Symbiotic bacteria system of *locusta migratoria* showed antifungal capabilities against *Beauveria bassiana*. *Int J Mol Sci* 2023;24:3138. 10.3390/ijms24043138 36834550 PMC9965112

[75] De Palma G, Shimbori C, Reed DE. et al. Histamine production by the gut microbiota induces visceral hyperalgesia through histamine 4 receptor signaling in mice. *Sci Transl Med* 2022;14:eabj1895. 10.1126/scitranslmed.abj1895 35895832

[76] Volonté C, Liguori F, Amadio S. A closer look at histamine in *drosophila*. *Int J Mol Sci* 2024;25:4449. 10.3390/ijms25084449 38674034 PMC11050612

[77] El Kholy S, Al NY. Insights into the mechanism of histamine synthesis and recycling disruption induced by exposure to CdO NPs in the fruit fly (*Drosophila melanogaster*). *Environ Sci Pollut Res Int* 2023;30:83376–87. 10.1007/s11356-023-28211-7 37340164

[78] Oh Y, Jang D, Sonn JY. et al. Histamine-HisCl1 receptor axis regulates wake-promoting signals in *Drosophila melanogaster*. *PLoS One* 2013;8:e68269. 10.1371/journal.pone.0068269 23844178 PMC3700972

[79] Luu P, Zaki SA, Tran DH. et al. A novel gene controlling the timing of courtship initiation in *Drosophila melanogaster*. *Genetics.* 2016;202:1043–53. 10.1534/genetics.115.183061 26721856 PMC4788109

[80] Hong ST, Bang S, Paik D. et al. Histamine and its receptors modulate temperature-preference behaviors in *drosophila*. *J Neurosci* 2006;26:7245–56. 10.1523/JNEUROSCI.5426-05.2006 16822982 PMC6673956

[81] Romanishina TA, Lakhman AR, Galatiuk OY. et al. Study of disinfectant activity against bee pathogenic enterobacteria in vitro. *Ukrainian Journal of Veterinary and Agricultural Sciences* 7:41–5. 10.32718/ujvas7-1.07

[82] Yuasa HJ, Ushigoe A, Ball HJ. Molecular evolution of bacterial indoleamine 2,3-dioxygenase. *Gene.* 2011;485:22–31. 10.1016/j.gene.2011.06.002 21689736

[83] Badawy AA . Kynurenine pathway of tryptophan metabolism: regulatory and functional aspects. *Int J Tryptophan Res* 2017;10:1178646917691938. 10.1177/1178646917691938 28469468 PMC5398323

[84] Bonaccorso S, Marino V, Puzella A. et al. Increased depressive ratings in patients with hepatitis C receiving interferon-alpha-based immunotherapy are related to interferon-alpha-induced changes in the serotonergic system. *J Clin Psychopharmacol* 2002;22:86–90. 10.1097/00004714-200202000-00014 11799348

[85] Maes M, Leonard BE, Myint AM. et al. The new '5-HT' hypothesis of depression: cell-mediated immune activation induces indoleamine 2,3-dioxygenase, which leads to lower plasma tryptophan and an increased synthesis of detrimental tryptophan catabolites (TRYCATs), both of which contribute to the onset of depression. *Prog Neuro-Psychopharmacol Biol Psychiatry* 2011;35:702–21. 10.1016/j.pnpbp.2010.12.017 21185346

[86] Kaur H, Bose C, Mande SS. Tryptophan metabolism by gut microbiome and gut-brain-Axis: an *in silico* analysis. *Front Neurosci* 2019;13:1365. 10.3389/fnins.2019.01365 31920519 PMC6930238

[87] Li Y, Hu N, Yang D. et al. Regulating the balance between the kynurenine and serotonin pathways of tryptophan metabolism. *FEBS J* 2017;284:948–66. 10.1111/febs.14026 28118532

[88] Eriksson M, Fowler CJ. Inhibition of monoamine oxidase and semicarbazide-sensitive amine oxidase by mexiletine and related compounds. *Naunyn Schmiedeberg's Arch Pharmacol* 1984;327:273–8. 10.1007/BF00506236 6514011

[89] Shih JC, Chen K, Ridd MJ. Monoamine oxidase: from genes to behavior. *Annu Rev Neurosci* 1999;22:197–217. 10.1146/annurev.neuro.22.1.197 10202537 PMC2844879

[90] Edmondson DE, Binda C. Monoamine oxidases. *Subcell Biochem* 2018;87:117–39. 10.1007/978-981-10-7757-9_5 29464559

[91] Blau N, Pearson TS, Kurian MA. et al. Aromatic L-amino acid decarboxylase deficiency. In: Adam M.P., Bick S., Mirzaa G.M.. et al. (eds.), GeneReviews®. Seattle (WA): University of Washington, Seattle, 2023. 37824694

[92] Han SW, Shin JS. Aromatic L-amino acid decarboxylases: mechanistic features and microbial applications. *Appl Microbiol Biotechnol* 2022;106:4445–58. 10.1007/s00253-022-12028-4 35763068

[93] Rahmdel S, Luqman A, Götz F. Microbiota-derived aromatic amino acid decarboxylases: linking microbial fitness and host neurochemical communication. *mBio.* 2025;16:e0205225. 10.1128/mbio.02052-25 41036868 PMC12607914

[94] Kumar S, Suleski M, Craig JM. et al. TimeTree 5: an expanded resource for species divergence times. *Mol Biol Evol* 2022;39:msac174. 10.1093/molbev/msac174 PMC9400175 35932227

[95] Hartenstein V, Martinez P. Structure. Development and evolution of the digestive system. *Cell Tissue Res* 2019;377:289–92. 10.1007/s00441-019-03102-x 31478136 PMC6820690

[96] Holtof M, Lenaerts C, Cullen D. et al. Extracellular nutrient digestion and absorption in the insect gut. *Cell Tissue Res* 2019;377:397–414. 10.1007/s00441-019-03031-9 31037358

[97] Toyofuku M, Inaba T, Kiyokawa T. et al. Environmental factors that shape biofilm formation. *Biosci Biotechnol Biochem* 2016;80:7–12. 10.1080/09168451.2015.1058701 26103134

[98] Aida H, Ying BW. Data-driven discovery of the interplay between genetic and environmental factors in bacterial growth. *Commun Biol* 2024;7:1691. 10.1038/s42003-024-07347-3 39719455 PMC11668901

[99] Smith A, Kaczmar A, Bamford RA. et al. The culture environment influences both gene regulation and phenotypic heterogeneity in *Escherichia coli*. *Front Microbiol* 2018;9:1739. 10.3389/fmicb.2018.01739 30158905 PMC6104134

[100] Roncarati D, Vannini A, Scarlato V. Temperature sensing and virulence regulation in pathogenic bacteria. *Trends Microbiol* 2025;33:66–79. 10.1016/j.tim.2024.07.009 39164134

