# Correction to: Behavioural phase transitions in the migratory locust, *Locusta migratoria*, are related to changes in the gut bacterial composition

**DOI:** 10.1093/ismeco/ycag235

**Published:** 2026-09-17

This is a correction to: Jaeha Kim, Takumi Murakami, Atsushi Toyoda, Hiroshi Mori, Behavioural phase transitions in the migratory locust, *Locusta migratoria*, are related to changes in the gut bacterial composition, *ISME Communications*, Volume 6, Issue 1, January 2026, ycag009, https://doi.org/10.1093/ismeco/ycag009

In the originally published version of this manuscript, the values in the ‘Total contig length (bp)’ column in Table 1 were missing their first digits.

Table 1 has been corrected to read:

**Table 1:** Summary of high-quality MAGs (completeness >90%, contamination <5%) showing the representative MAG with the highest completeness and lowest contamination for each species.

Code of the host	Type of the host	Species name	Contigs number	GC(%)	N50	Total contig length (bp)	CDS	Completeness (%)	Contamination (%)
Lab-23-Sol-Female-2	Gregarious	*Serratia ureilytica*	46	59.5	175 940	5,067,132	4702	97.21	1.068
Lab-23-Sol-Female-2	Gregarious	*Klebsiella aerogenes*	50	55.1	189 738	5,015,506	4633	97.99	1.006
Lab-23-Sol-Female-2	Gregarious	*Enterobacter hormaechei B*	49	55.7	164 223	4,438,642	4104	96.99	0.308
Lab-23-Sol-Male-3	Solitary	*Acinetobacter oleivorans*	32	38.5	230 827	3,929,983	3650	100	0.684
Lab-23-Sol-Female-1	Solitary	*Stenotrophomonas geniculata*	123	66.6	52 535	4,381,003	3989	98.03	0
Lab-23-Sol-Female-1	Gregarious	*Pantoea latae*	70	59.8	113 294	4,492,408	4100	90.53	0.402
Lab-23-Gre-Male-4	Gregarious	*Pantoea ananatis*	107	53.4	112 647	4,736,562	4389	93.03	1.315
Lab-23-Sol-Female-2	Solitary	*Pantoea endophytica*	60	54.8	130 066	4,687,411	4263	93.29	0
Wild-22-Kise-Female-3	Wild	*Xanthomonas albilineans*	96	63.1	71 461	3,502,053	2943	99.44	0.258
Wild-22-Kise-Male-2	Wild	*Spiroplasma phoeniceum*	236	26.6	6651	1,032,578	1875	93.58	0

Instead of:

**Table 1:** Summary of high-quality MAGs (completeness >90%, contamination <5%) showing the representative MAG with the highest completeness and lowest contamination for each species.

Code of the host	Type of the host	Species name	Contigs number	GC(%)	N50	Total contig length (bp)	CDS	Completeness (%)	Contamination (%)
Lab-23-Sol-Female-2	Gregarious	Serratia ureilytica	46	59.5	175 940	,067132	4702	97.21	1.068
Lab-23-Sol-Female-2	Gregarious	Klebsiella aerogenes	50	55.1	189 738	,015506	4633	97.99	1.006
Lab-23-Sol-Female-2	Gregarious	Enterobacter hormaechei *B*	49	55.7	164 223	,438 642	4104	96.99	0.308
Lab-23-Sol-Male-3	Solitary	*Acinetobacter oleivorans*	32	38.5	230 827	,929 983	3650	100	0.684
Lab-23-Sol-Female-1	Solitary	*Stenotrophomonas geniculata*	123	66.6	52 535	,381 003	3989	98.03	0
Lab-23-Sol-Female-1	Gregarious	*Pantoea latae*	70	59.8	113 294	,492 408	4100	90.53	0.402
Lab-23-Gre-Male-4	Gregarious	Pantoea ananatis	107	53.4	112 647	,736 562	4389	93.03	1.315
Lab-23-Sol-Female-2	Solitary	*Pantoea endophytica*	60	54.8	130 066	,687 411	4263	93.29	0
Wild-22-Kise-Female-3	Wild	Xanthomonas albilineans	96	63.1	71 461	,502 053	2943	99.44	0.258
Wild-22-Kise-Male-2	Wild	Spiroplasma phoeniceum	236	26.6	6651	,032578	1875	93.58	0

