# Supplementary material for: Behavioural phase transitions in the migratory locust, Locusta migratoria, are related to changes in the gut bacterial composition
Source: ISME Commun. 2026 Jan 15;6(1):ycag009. doi: 10.1093/ismeco/ycag009 (PMC12903957; doi:10.1093/ismeco/ycag009)
Supplement: Figure_S1_revision_ycag009 [file figure_s1_revision_ycag009.pdf]

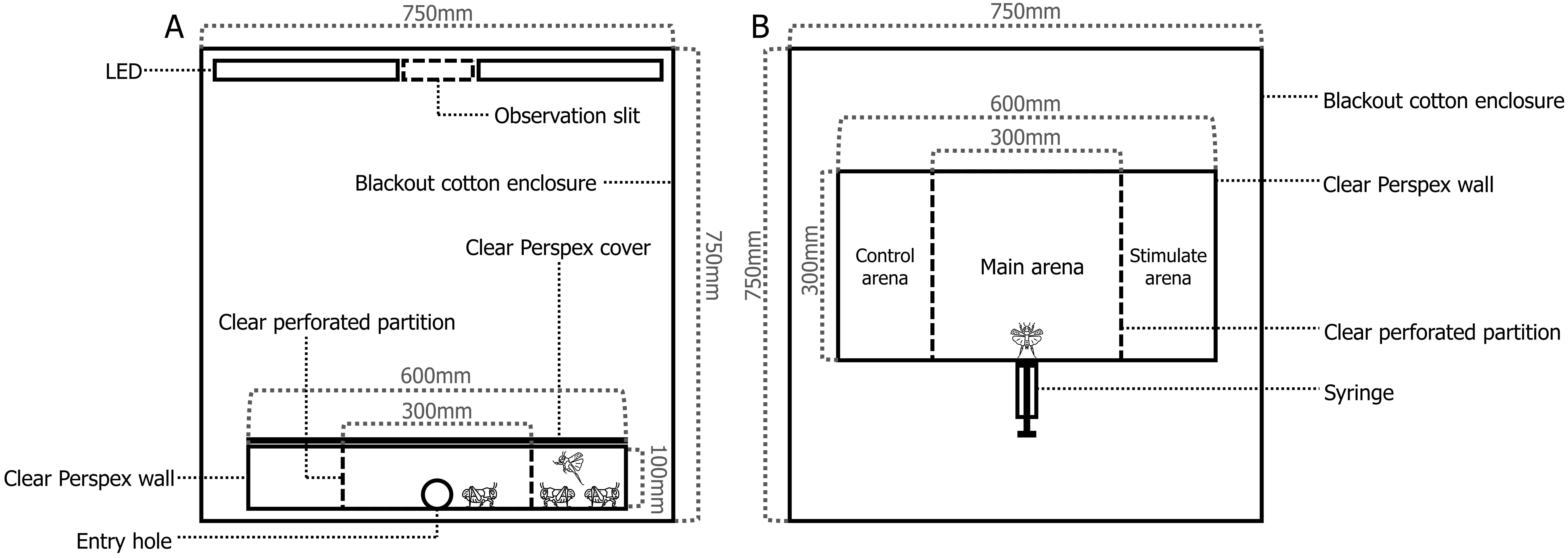
A
750mm
LED
Observation slit
Blackout cotton enclosure
750mm
Clear Perspex cover
Clear perforated partition
600mm
300mm
100mm
Clear Perspex wall
Entry hole
B
750mm
Blackout cotton enclosure
600mm
300mm
Clear Perspex wall
750mm
300mm
Control arena
Main arena
Stimulate arena
Clear perforated partition
Syringe
