# Supplementary figures and images for: Behavioural phase transitions in the migratory locust, Locusta migratoria, are related to changes in the gut bacterial composition
Source: ISME Commun. 2026 Jan 15;6(1):ycag009. doi: 10.1093/ismeco/ycag009 (PMC12903957; doi:10.1093/ismeco/ycag009)

A

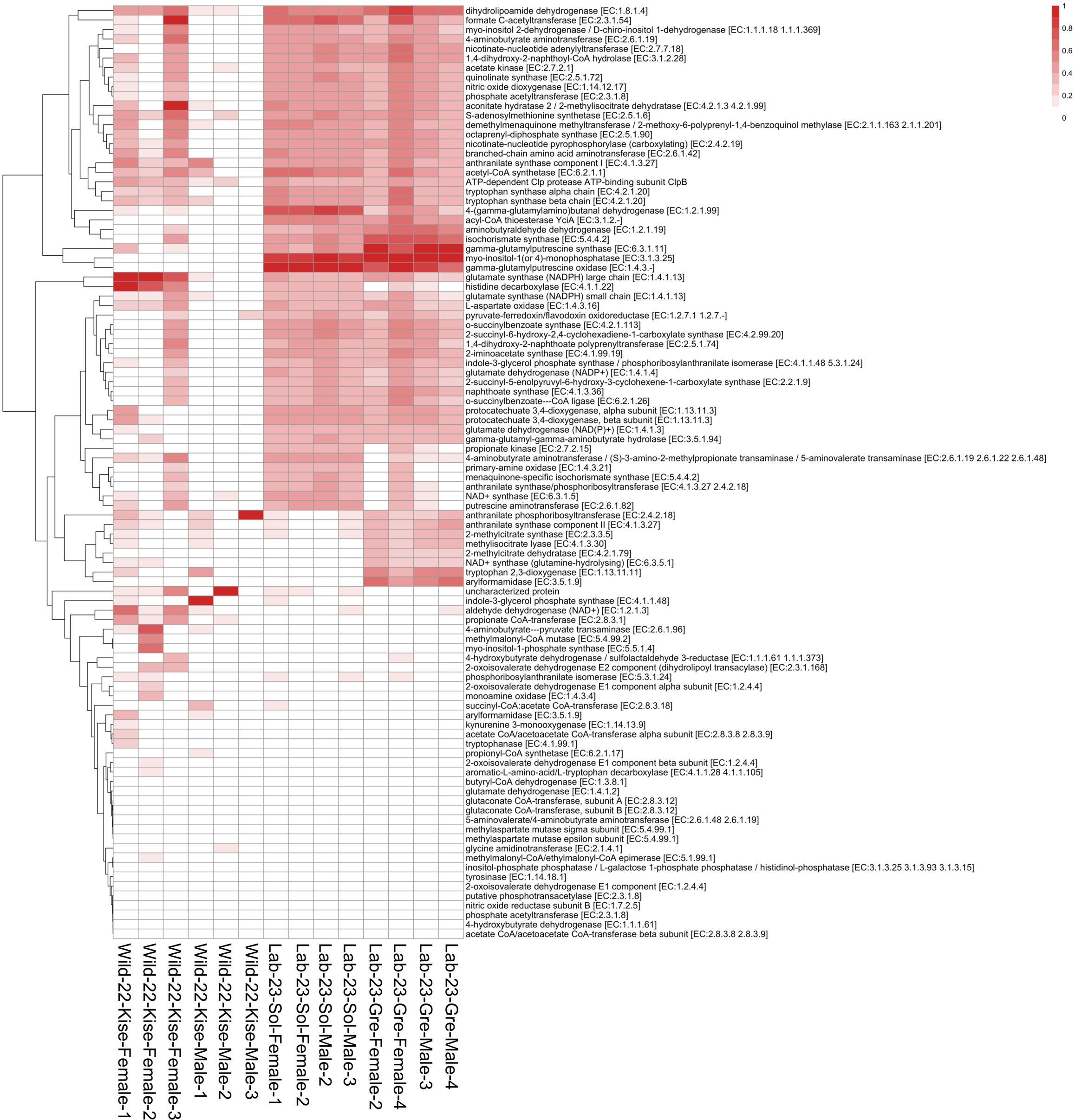

Supplement: Figure_S2_revision_ycag009 [file figure_s2_revision_ycag009.pdf]
