# Supplementary material for: Behavioural phase transitions in the migratory locust, Locusta migratoria, are related to changes in the gut bacterial composition
Source: ISME Commun. 2026 Jan 15;6(1):ycag009. doi: 10.1093/ismeco/ycag009 (PMC12903957; doi:10.1093/ismeco/ycag009)
Supplement: supplementary_material_revision_20251231_ycag009 [file supplementary_material_revision_20251231_ycag009.docx]

**Supplementary Method**

**Modified DNA extraction protocol for faecal samples of the grasshopper**

400 mg of the faecal sample from the same individual were divided into eight 1.5 ml microcentrifuge tubes of 50 mg each and placed on ice. 500 μl of InhibitEX Buffer was added to each tube and vortexed vigorously for two minutes. All mixtures were incubated for five min at 70°C and vortexed for 15 sec. All mixtures were centrifuged at full speed (16,000 × g) for 1 min. 30 μl of Proteinase K was pipetted in two new 1.5 ml microcentrifuge tubes. 100 μl of supernatant from each centrifuged 1.5 ml tube were pipetted into the 1.5 ml microcentrifuge tube containing Proteinase K until two new 1.5 ml tubes contained 400 μl of supernatant each. 400 μl of Buffer AL was added into the 1.5 ml tube containing 415 μl of mixture and vortexed for 15 s. All mixtures were left for ten min at 70°C and 400 μl of ethanol (95%) was added into each tube. After vortexing for 15 s, 600 μl of the mixture was carefully applied to the QIAamp spin column and centrifuged for one minute. After centrifuge, the QIAamp spin column was placed in a new 2 ml collection tube, and the tube containing the filtrate was discarded. These centrifuge steps were repeated until all the mixture was loaded with only one QIAamp spin column. The QIAamp column was washed with 500 μl of Buffer AW1, and washed again with 500 μl of Buffer AW2. After washing, the QIAamp column was placed in a new labeled 1.5 ml microcentrifuge tube and 25-50 μl of AE Buffer was directly applied onto the QIAamp membrane. A 1.5 ml spin column placed microcentrifuge tube was incubated for one minute at room temperature, and then centrifuged for one minute to elute DNA. DNA eluted solutions were kept at -30°C until the sequencing.

**Modified behavioural analysis method to discriminate behavioural phase of *L. migratoria***

The behavioural analysis method was used to distinguish the behavioural phase of *L. migratoria* by referring to previously published studies [1-3]. The assay arena (Supplementary Figure S1) was designed to statistically record the behavioural characteristics of *L. migratoria*. All parts of the assay arena were made with clear Perspex only except a white grid-marked filter paper floor. Each area was divided with a clear perforated Perspex partition so that individuals located on the main stage were able to see and smell the stimulation area and control area, and could avoid or approach them. The assay arena was located inside the blackout cotton enclosure so that the outside movement of an observer could not give any stimulus to the test individual. A camera was located on the observation slit to record all movements of the test individual.

Before the test, 35 individuals of high-density reared adult *L. migratoria* were placed in the stimulus arena with fresh silver grass leaves, while the control arena only contained fresh silver grass leaves. A test individual was placed and left in a blackened plastic syringe connected main area for 300 sec. After that, a test individual was introduced into the main arena by pushing the syringe. All movements of the test individuals were recorded by camera for 500 sec. All observation experiments were conducted between 10 a.m. to 4 p.m. in consideration that *L. migratoria* is a diurnal insect. Behavioural parameters used in previously published studies [1-3] were measured, namely: walking speed (cm/s), fraction of time spent motionless (motionless time/500), time spent in the 25% area of the main arena next to the stimulus area (s), frequency of grooming (grooming number/s), Attraction index (total duration in stimulus area – total duration in the opposite of stimulus area), total distance moved (cm), and total duration of movement (s). To analyze walking speed and total distance moved, a video analysis program Duomouse (Arakawa et al. 2014) [4] was used, while other parameters were analyzed manually. Behavioural covariates and parameters were referred from a previous study of *L. migratoria* (Le Kang et al. 2011) [3]. *P*_greg_ *value* was calculated using a formula, 𝑃_greg_ = 𝑒^η^/(1+ 𝑒^η^), η = -2.11 + 0.005 × Attraction index + 0.012 × total distance moved + 0.015 × total duration of movement.

**Sample ID naming pattern**

Each individual was assigned a unique sample ID consisting of six components: [environment code]-[collection year]-[sample type code]-[sex code]-[serial number]-[age code]. The purpose of this system was to systematically capture metadata about the origin and characteristics of each sample. The definitions of each component are as follows:

- Environment code: Indicates the sampling environment. Two environments were analysed, the wild environment (“Wild”) and the laboratory environment (“Lab”).
- Collection year: The year in which the sample was collected (e.g., “23” for 2023).
- Sample type code: Provides more specific information about sample’s origin. For wild-type samples, this code indicates the sampling location: the Kisewaga area (“Kise”) and the Tamagawa area (“Tama”). For laboratory-rared samples, this code indicates the behavioural phase: the gregarious phase (“Gre”) and the solitary phase (“Sol”).
- Sex code: Indicates the sex of the individual: “Female” or “Male”. For gregarious-phase nymphs collected as a group, the sex code was described as "Group".
- Serial number: A numerical identifier to distinguish individuals within the same category group. For gregarious-phase nymph samples with “Group” in their sex code, this serial numbers indicate independent biological duplicates prepared under identical conditions. Therefore, two samples with a different serial number, "Group-1" and "Group-2", are theoretically same.
- Age code: Indicates the developmental stage. This code was omitted for representative samples of adults collected three days after emergence or when the exact age was unknown. When present, the age code specifies the exact stage: “I” = first instar nymph, “II” = second instar, “III” = third instar, “IV” = fourth instar, “V” = fifth instar, “VI” = ten days after the adult emergence.

For example, the ID “Wild-23-Kise-Male-1” represents a wild-type individual sampled in 2023 at Kisegawa, male, and is the first sample collected. The absence of an age code indicates that this is a representative adult sample collected three days after emergence or with an unidentified age.

**Supplementary Figure legends**

**Figure S1.** The assay arena blueprint. (A) Side and (B) top view of the assay arena for assessing the behavioural recording. A similar experiment was described from the *S. gregaria* study [5], but modified for *L. migratoria*.

**Figure S2.** (A) A heatmap of the annotated GBM associated KO gene abundances based on the TPM values. Each KO ID was transferred to its enzyme function and EC number. Red means high abundance in the metagenomic sample.

**Supplementary Table legends**

**Supplementary Table S1.** Behavioural parameters and results of the *t*-test of laboratory-reared type gregarious and solitary *Locusta migratoria* in 2023

**Supplementary Table S2.** Behavioural parameters and results of the *t*-test of laboratory-reared type gregarious and solitary *Locusta migratoria* in 2024

**Supplementary Table S3.** Assembly statistics of shotgun metagenomic sequencing analysis.

**Supplementary Table S4.** The taxonomic composition of the gut microbiota using shotgun metagenomic sequencing.

**Supplementary Table S5.** ASV table of the 16S rRNA gene amplicon sequencing analysis.

**Supplementary Table S6.** List of all identified KEGG Orthologies in faecal samples of *Locusta migratoria*, and TPM values in each sample. Differential-abundance results are summarized in the column “Log₂ fold change between solitary (−) and gregarious (+)”, and in “Adjusted p-value (FDR)”. The final column, “Statistical significancy,” indicates whether each KO is significantly enriched in solitary or gregarious samples. An asterisk (*) denotes an FDR below 0.05, indicating a statistically significant difference between the two groups. When the asterisk is followed by the name of a specific group, it signifies that the absolute log₂ fold change exceeds 1, indicating that the KO is significantly enriched in that group. In contrast, an asterisk without a group designation indicates a significant difference between groups, but with no enrichment in either group.

**Supplementary Table S7.** Metabolite pathways, including GBMs, and associated KOs showing differences between solitary and gregarious samples. The column “Number of metabolic steps” specifies how many biochemical reactions constitute the pathway, and “Number of necessary KOs” indicates the minimum number of KOs required to complete the pathway. A logical expression of KO IDs that collectively represent all enzymatic steps of the pathway is indicated in the column “KEGG Orthology composition of pathway”. For each KO, the table reports its identifier in the column “KO ID,” the corresponding functional annotation in “Function of KEGG Orthology,” and the biochemical step at which it operates in “Step number in pathway”. The subsequent columns–from “Lab-23-Sol-Male-2” to “Lab23-Gre-Female-4”–present the TPM value of each KO in the corresponding sample. Differential-abundance results are summarized in the column “Log₂ fold change between solitary (−) and gregarious (+)”, and in “Adjusted p-value (FDR)”. The final column, “Statistical significancy,” indicates whether each KO is significantly enriched in solitary or gregarious samples. An asterisk (*) denoted an FDR below 0.05, indicating a statistically significant difference between the two groups. When the asterisk is followed by the name of a specific group, it indicates that the absolute log₂ fold change exceeds 1 and that the KO is significantly enriched in that group. Conversely, an asterisk without a group designation indicates a significant difference between the two groups but without enrichment in either group. “N.A.” indicates that the statistical test was not applicable.

**Supplementary Table S8.** Summary of all constructed high-quality MAGs.

**Supplementary Table S9**. Metabolite pathways, including GBMs, and associated KOs identified in representative MAGs of ten species. The column “Number of metabolic steps” specifies how many biochemical reactions constitute the pathway, and “Number of necessary KOs” indicates the minimum number of KOs required to complete the pathway. A logical expression of KO IDs that collectively represent all enzymatic steps of the pathway is indicated in the column “KEGG Orthology composition of pathway”. For each KO, the table reports its identifier in the column “KO ID,” the corresponding functional annotation in “Function of KEGG Orthology,” and the biochemical step at which it operates in “Step number in pathway”. The subsequent columns–from “*Serratia ureilytica*” to “*Xanthomonas albilineans*”–present whether each KO has been identified in the corresponding MAG.

**References**

1) Simpson SJ, Despland E, Hägele BF, Dodgson T. Gregarious behavior in desert locusts is evoked by touching their back legs. *Proc Natl Acad Sci U S A*. 2001;98(7):3895-3897. doi:10.1073/pnas.071527998

2) Rogers SM, Cullen DA, Anstey ML, Burrows M, Despland E, Dodgsom T, Matheson T, Ott SR, Stettin K, Sword GA, Simpson SJ. et al. Rapid behavioural gregarization in the desert locust, *Schistocerca gregaria* entails synchronous changes in both activity and attraction to conspecifics. *J Insect Physiol*. 2014;65(100):9-26. doi:10.1016/j.jinsphys.2014.04.004

3) Guo W, Wang X, Ma Z, et al. CSP and takeout genes modulate the switch between attraction and repulsion during behavioral phase change in the migratory locust. *PLoS Genet*. 2011;7(2):e1001291. doi:10.1371/journal.pgen.1001291

4) Arakawa T, Tanave A, Ikeuchi S, Takahashi A, Kakihara S, Kimura S, Sugimoto H, Asada N, Shiroishi T, Tomihara K, Tsuchiya T, Koide T. A male-specific QTL for social interaction behavior in mice mapped with automated pattern detection by a hidden Markov model incorporated into newly developed freeware. *J Neurosci Methods*. 2014;234:127-134. doi:10.1016/j.jneumeth.2014.04.012

5) Roessingh, P., Simpson, S. J., & James, S. Analysis of Phase-Related Changes in Behaviour of Desert Locust Nymphs. *Biological Sciences*. 1993;252(1333):43–49. doi:10.1098/rspb.1993.0044
